# Comprehensive understanding of Tn5 insertion preference improves transcription regulatory element identification

**DOI:** 10.1093/nargab/lqab094

**Published:** 2021-10-27

**Authors:** Houyu Zhang, Ting Lu, Shan Liu, Jianyu Yang, Guohuan Sun, Tao Cheng, Jin Xu, Fangyao Chen, Kuangyu Yen

**Affiliations:** State Key Laboratory of Experimental Hematology, National Clinical Research Center for Blood Diseases, Institute of Hematology and Blood Diseases Hospital, Chinese Academy of Medical Sciences and Peking Union Medical College, Tianjin 300020, China; School of Biology and Biological Engineering, South China University of Technology, Guangzhou 510006, China; State Key Laboratory of Experimental Hematology, National Clinical Research Center for Blood Diseases, Institute of Hematology and Blood Diseases Hospital, Chinese Academy of Medical Sciences and Peking Union Medical College, Tianjin 300020, China; State Key Laboratory of Experimental Hematology, National Clinical Research Center for Blood Diseases, Institute of Hematology and Blood Diseases Hospital, Chinese Academy of Medical Sciences and Peking Union Medical College, Tianjin 300020, China; School of Biology and Biological Engineering, South China University of Technology, Guangzhou 510006, China; Department of Developmental Biology, School of Basic Medical Sciences, Southern Medical University, Guangzhou 510515, China; State Key Laboratory of Experimental Hematology, National Clinical Research Center for Blood Diseases, Institute of Hematology and Blood Diseases Hospital, Chinese Academy of Medical Sciences and Peking Union Medical College, Tianjin 300020, China; State Key Laboratory of Experimental Hematology, National Clinical Research Center for Blood Diseases, Institute of Hematology and Blood Diseases Hospital, Chinese Academy of Medical Sciences and Peking Union Medical College, Tianjin 300020, China; Division of Cell, Developmental and Integrative Biology, School of Medicine, South China University of Technology, Guangzhou 510006, China; Department of Epidemiology and Biostatistics, School of Public Health, Xi’an Jiaotong University Health Science Center, Xi’an, Shaanxi 710061, China; State Key Laboratory of Experimental Hematology, National Clinical Research Center for Blood Diseases, Institute of Hematology and Blood Diseases Hospital, Chinese Academy of Medical Sciences and Peking Union Medical College, Tianjin 300020, China; Department of Developmental Biology, School of Basic Medical Sciences, Southern Medical University, Guangzhou 510515, China

## Abstract

Tn5 transposase, which can efficiently tagment the genome, has been widely adopted as a molecular tool in next-generation sequencing, from short-read sequencing to more complex methods such as assay for transposase-accessible chromatin using sequencing (ATAC-seq). Here, we systematically map Tn5 insertion characteristics across several model organisms, finding critical parameters that affect its insertion. On naked genomic DNA, we found that Tn5 insertion is not uniformly distributed or random. To uncover drivers of these biases, we used a machine learning framework, which revealed that DNA shape cooperatively works with DNA motif to affect Tn5 insertion preference. These intrinsic insertion preferences can be modeled using nucleotide dependence information from DNA sequences, and we developed a computational pipeline to correct for these biases in ATAC-seq data. Using our pipeline, we show that bias correction improves the overall performance of ATAC-seq peak detection, recovering many potential false-negative peaks. Furthermore, we found that these peaks are bound by transcription factors, underscoring the biological relevance of capturing this additional information. These findings highlight the benefits of an improved understanding and precise correction of Tn5 insertion preference.

## INTRODUCTION

The rapid rise in DNA sequencing capacity, particularly short-read sequencing, has created a growing need for affordable, simple methods to prepare sequencing libraries. To streamline library preparation, the Tn5 transposase has been modified to create a hyperactive version that can efficiently tagment the genome: a combined enzymatic activity that achieves fragmentation and adaptor ligation in a single step (1–4). Tn5 is now used in a number of genomic sequencing technologies (see Supplementary Table S1 for a summary).

One of these additional sequencing technologies is assay for transposase-accessible chromatin using sequencing (ATAC-seq) (5). ATAC-seq measures chromatin accessibility via Tn5 insertion along the chromatin either in the bulk population or at the single-cell level (5,6). The primary application of ATAC-seq is to identify potential cis-regulatory elements (CREs), providing a genome-wide view of transcriptional regulation (7–9). Besides chromatin accessibility, ATAC-seq has been used to infer transcription factor (TF) occupancy by analyzing the shape and altitude of Tn5 insertion profiles, a technique termed digital footprinting analysis (5). ATAC-seq footprinting has been successfully applied to predict the regulatory networks during development and in cancer (8,10–13). Although previous studies reported that Tn5 inserts into the genome in a near-random pattern, with negligible sequence preference (4,14,15), it was recently observed that even on naked genomic DNA, Tn5 could produce ‘pseudo-TF footprints’ (10,16), suggesting that Tn5 has some intrinsic insertion preference.

Tn5 insertion preference was initially studied by Berg *et al.* (14), whose work showed that a G/C pair frequently occurs at the edges of a 9-bp region surrounding insertion sites. However, several subsequent studies reported conflicting preferences for GC-rich (17–19) or AT-rich regions (20,21) based on a large number of Tn5 insertion sites. In addition to GC content, it was recently reported that DNA shape affects Tn5 insertion in HepG2 chromatin, whereas DNA methylation has minimal impact on Tn5 insertion (22). These results, from studies that were done in different species or chromatin contexts, and used different evaluation methods, highlight the need for systematic mapping of Tn5 insertion and a more complete understanding of its insertion bias(es).

Moreover, because Tn5 bias has not been comprehensively evaluated, current Tn5 bias correction methods are largely adapted from protocols developed for analyzing DNase digital footprinting. DNase cleavage bias, however, is distinct from that of Tn5 (23–25). It is perhaps not surprising then that, when using correction methods, the footprinting performance for ATAC-seq has been reported as less satisfactory compared with DNase-seq (24,26,27). Furthermore, although correction for Tn5 insertion bias is a prerequisite for digital footprinting, whether bias correction benefits general ATAC-seq applications, such as the widely adopted peak calling analysis framework, has not been evaluated.

Here, we systematically studied Tn5 insertion preference across eight model organisms in naked genomic DNA and chromatin contexts. We studied the influence of intrinsic parameters (DNA motif and DNA shape) and an epigenomic parameter (DNA methylation) on Tn5 insertion preference. Using this information, we developed a computational bias correction pipeline. We showed that bias correction improves the performance of peak calling algorithms. Moreover, we find that bias correction identifies regions enriched for TFs that were not called as peaks using traditional peak calling methods. Thus, another benefit of performing Tn5 insertion bias correction is the reduction of false negatives in ATAC-seq data analysis.

## MATERIALS AND METHODS

### Cell culture and primary cell acquisition

Primary hematopoietic stem and progenitor cells (HSPCs) were obtained as described in (28). Briefly, cKit magnetic beads (Miltenyi Biotec) were used to enrich HSPCs from mouse bone marrow and cultured in IMDM (Gibco) supplemented with 15% FBS (Gibco), 10 ng/ml mIL-6, 10 ng/ml mIL-3, 50 ng/ml SCF, 20 ng/ml thrombopoietin (PeproTech) and 10 ng/ml Flt3 ligand (PeproTech).

The E14 and K562 cell lines were obtained from the experimental pathology cell bank in State Key Laboratory of Experimental Hematology. The K562 cells were cultured in RPMI-1640 (Gibco) supplemented with 10% FBS. E14 cells were maintained in 0.2% gelatin (Sigma)-coated plates in 2i medium, which consisted of DMEM/F12 supplemented with Neurobasal medium, serum-free N2B27 medium supplemented with 10 μM MEK inhibitor PD0325901 and 30 μM GSK3 inhibitor CHIR99021 (both from Selleckchem), 2% KnockOut™ serum replacement (Gibco), 0.002% BSA (Gibco), 1 mM MTG (Gibco), 1000 U/ml LIF (Millipore), 0.1 mM non-essential amino acid and 2 mM GlutaMAX Supplement (Gibco).

### Tagmentation and sequencing of naked genomic DNA

DNA was purified with a QIAamp DNA Mini Kit (QIAGEN), and 50 ng DNA was used for the following procedure. After DNA extraction, we used a Thermo Scientific NanoDrop 2000 Spectrophotometer to measure 260/280 and 260/230 ratios to confirm DNA purity. The DNA was added to a 50 μl transposition reaction mix (5 μl TruePrep Tagment Enzyme, 10 μl TruePrep Tagment Buffer L and 35 μl ddH_2_O from Vazyme TD501-01) followed by incubation at 55°C for 10 min. After tagmentation, VAHTS DNA Clean Beads were used to stop the reaction, and DNA was purified for final library construction (TruePrep™ DNA Library Prep Kit V2 for Illumina) before paired-end high-throughput sequencing using an Illumina Next550 or NovaSeq 6000.

### Genome sequencing data preprocessing

The genome FASTA sequence and GTF annotation files were downloaded from the Ensembl database (29). Genome assembly versions for each species are as follows: mouse (*Mus musculus*, *mm10*), human (*Homo sapiens*, *hg38*), nematode (*Caenorhabditis elegans*, *ce11*), fruit fly (*Drosophila melanogaster*, *dm6*), zebrafish (*Danio rerio*, *danRer11*), *Plasmodium* (*Plasmodium falciparum*, *pfa2*), *Arabidopsis* (*Arabidopsis thaliana*, *tair10*) and maize (*Zea mays*, *zm3*).

Raw fastq data were parsed using the SRA Explorer website (https://github.com/ewels/sra-explorer) from the Gene Expression Omnibus (30) and Encyclopedia of DNA Elements (ENCODE) (31) database and downloaded using Aspera version 3.9.8.176272 (https://www.ibm.com/products/aspera). Detailed information including accession numbers and sample information is available in Supplementary Table S2.

FastQC (http://www.bioinformatics.babraham.ac.uk/-projects/fastqc/) version 0.11.5 was used to check the sequencing quality of raw fastq data. Trimmomatic version 0.33.0 (32) was used to trim protocol-specific sequencing adapters. Bedtools version 2.29.1 (33) and Bedtk version 0.0-r24 (34) were used in this study for bed format file manipulation.

### ATAC-seq data processing

Bowtie2 version 2.2.7 (35) was used for mapping reads to their respective reference genomes using the ‘--end-to-end --no-mixed -X 2000’ parameter, where ‘-X 2000’ allows the maximum fragment length to be 2000 bp and ‘--no-mixed’ suppresses unpaired alignments for paired reads. To minimize PCR and sequencing optical bias, Picard (http://broadinstitute.github.io/picard) version 2.9.0 subcommand *MarkDuplicates* was used to mark duplicates (defined as the same start and end positions) and *CollectInsertSizeMetrics* was used to estimate fragment size distribution. Samtools version 1.7 (36) was used for SAM file manipulation. Specifically, subcommand *view* with ‘-F 1804 -q 30 -b’ parameter was used to remove low-quality, unmapped, unpaired and duplicated reads, as well as convert to BAM format. Subcommands *flagstat*, *sort* and *index* with default parameters were used to summarize, sort and index BAM files.

Because 9-bp target duplications are generated during Tn5 transposition (14), we shifted reads on the forward strand by +4 bp and reads on the reverse strand by −5 bp, following the instructions previously reported (5) to get the centers of Tn5 insertion events (aliased as Tn5 insertion sites). This single-base Tn5 insertion site information was used in bed, bam and BigWig formats for downstream analysis.

BigWig format files were generated via *bamCoverage* subcommand in deeptools2 (37) with the ‘--binSize 1’ parameter. Where available, ENCODE blacklists (38) including ‘High Signal Region’ and ‘Low Mappability Regions’ were excluded from downstream analysis.

The genome-wide Tn5 insertion sites in chromatin and naked genomic DNA of mouse embryonic stem cells (ESCs), HSPCs and human K562 cells were uploaded into the UCSC Genome Browser, which can be accessed through these track hubs: https://raw.githubusercontent.com/YenLab/Tn5InsertPrefer/main/UCSC_tracks/Mouse/hub.txt and https://raw.githubusercontent.com/YenLab/Tn5InsertPrefer/main/UCSC_tracks/Human/hub.txt.

### Mapping Tn5 insertions across genomic features

We measured the Tn5 insertion preference across genomic features using the ratio between observed and expected Tn5 insertion sites. Genomic features used in this study are classified into three categories:

1. *Basic genomic annotations*: transcription start sites (TSSs), transcription terminal sites, CpG island, introns, exons, intergenic and untranslated region.
2. *ENCODE3 candidate CREs* (7): distal/proximal enhancer-like signatures (d/pELS), distal/proximal promoter-like signatures (d/pPLS), CTCF and DNase–H3K4me3.
3. *Repetitive genomic features* from RepeatMasker (39): satellites, simple repeat, low-complexity regions, retrotransposons [long interspersed nuclear element (LINE), short interspersed nuclear element (SINE), and long terminal repeat (LTR)], rRNA and tRNA.

**Figure 1.**
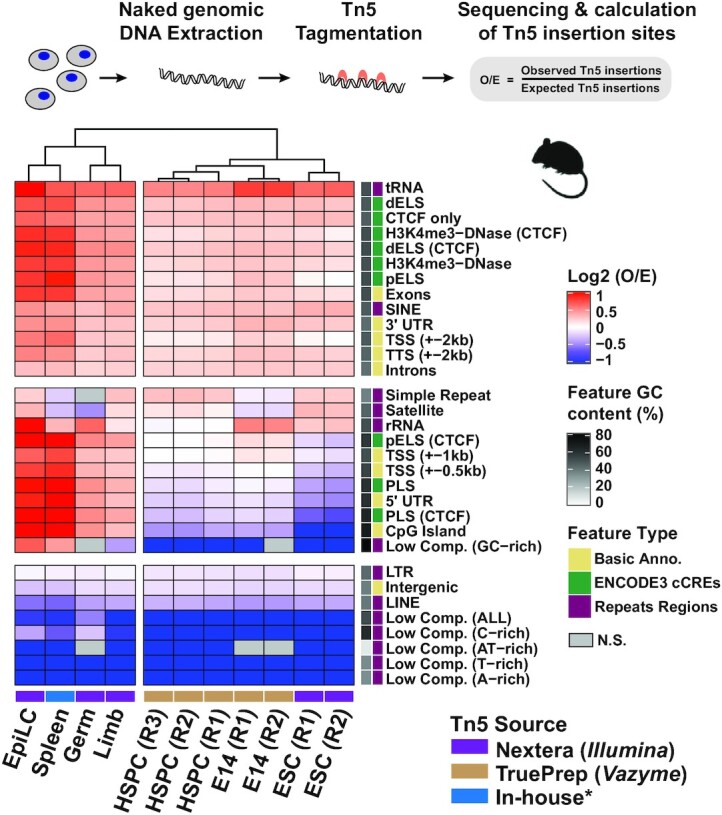
Tn5 does not insert randomly in naked genomic DNA. Distribution of Tn5 insertion sites across genomic features in mouse naked genomic DNA. Top panel: Schematic showing sequencing and calculation of Tn5 insertion sites. Bottom panel: Heatmap showing the distribution of Tn5 insertion sites. Red indicates the enrichment of Tn5 insertion sites; blue indicates depletion. Nonsignificant values (N.S.) are colored gray, with false discovery rate (FDR) < 0.001 as a threshold (chi-square test corrected by FDR). Cell types were clustered using the ‘complete’ algorithm. The Tn5 source for each sample is indicated, and ‘In-house*’ refers to Tn5 purified following the procedure in (1). E14 and HSPC data were generated in this study; public data for EpiLC (84), spleen (85), germ (48), limb (86) and ESCs (87) were used. EpiLC, epiblast-like cell; HSPC, hematopoietic stem and progenitor cell; ESC, embryonic stem cell; R, biological replicate; dELS or pELS, distal or proximal enhancer-like signature; PLS, promoter-like signature; CTCF only, only CTCF-bound regions; H3K4me3–DNase, both H3K4me3 and DNase I hypersensitive peaks occur in these regions; (CTCF) indicates CTCF bound in these regions (7); SINE, short interspersed nuclear element; LINE, long interspersed nuclear element; LTR, long terminal repeat; Low Comp., low-complexity regions, which were grouped based on the nucleotide content in each region, for example AT-rich and C-rich (39).

The ratio between observed and expected Tn5 insertion sites was represented by the log_2_(*O*/*E*) value, where *E* (expected insertion sites) was estimated by *total feature length ×* (*all Tn5 insertion sites/mappable genome size*) and *O* (observed insertion sites) was counted directly from features using deeptools *intersect* command. After log_2_ transformation, features enriched for Tn5 insertion sites were indicated by positive values and features depleted for Tn5 insertion sites were indicated by negative values; these data were plotted in the form of heatmap via *ComplexHeatmap* (40) (Figure 1; Supplementary Figure S2). The dendrogram of cell types was clustered using the ‘complete’ algorithm in the *hclust()* function from the base R package.

The *chi-squared test for goodness of fit* was used to determine whether there was a statistically significant difference between the expected and the observed Tn5 insertion in each feature of each sample. The FDR (41) was used to adjust the *P*-value and FDR < 0.001 was used as a threshold.

### Effect of DNA motif on Tn5 insertion

**Figure 2.**
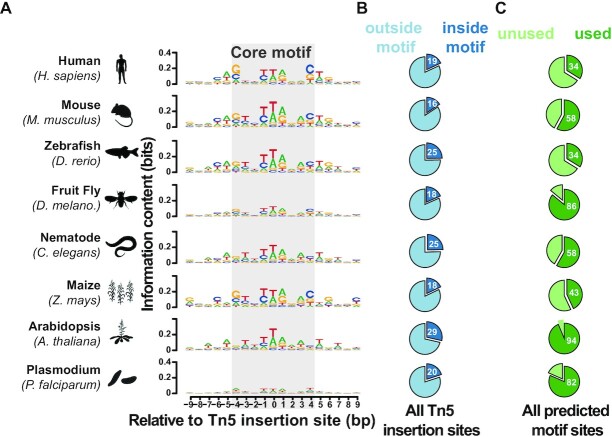
DNA motif signature is insufficient for explaining Tn5 insertion preference. (**A**) Motifs underlying Tn5 insertion sites in a range of species. Motifs were returned by MEME (42) using a 19-bp window around Tn5 insertion sites. The core 9-bp motif proposed initially in (14) is shaded gray. (**B**) All observed Tn5 insertion sites that overlap with the predicted motif were denoted as ‘inside motif’, otherwise they were denoted as ‘outside motif’. (**C**) All potential Tn5 motif site locations along the genome in each species were searched using FIMO (42) using the corresponding PWM in each species. Motifs that overlap observed Tn5 insertion sites were denoted as ‘Used’, otherwise they were denoted as ‘Unused’.

Large numbers of Tn5 insertion sites in NGS data might conceal noise, and different sources of data might introduce batch effects in the analysis. To address this, we searched the pool of Tn5 insertion sites unbiasedly for paired fragments, defined as exactly 9 bp of two adjacent fragments with reverse complementarity. We randomly sampled an equal size dataset (500 000) for each sample (Figure 2A). Based on these sites, a position weight matrix (PWM) showing the most favored nucleotide composition was returned by MEME (Multiple EM for Motif Elicitation) (42). To validate the PWM, potential Tn5 motifs for each species were predicted genome-wide using FIMO (Find Individual Motif Occurrences) (43) using the *q*-value <0.001 as a threshold. We calculated the fraction of predicted motifs overlapping Tn5 insertion sites and the fraction of Tn5 insertion sites that fall within motifs, which resulted in four categories (Figure 2B and C):

1. *insert inside*: the Tn5 insertion sites fall within predicted motifs.
2. *insert outside*: the Tn5 insertion sites fall outside predicted motifs.
3. *motif used*: the predicted motifs have Tn5 insertion.
4. *motif unused*: the predicted motifs have no Tn5 insertion.

### Effect of DNA shape on Tn5 insertion

#### DNA shape calculation

In this study, we used the DNA shapes defined in (44), including 14 types that can be calculated using the DNAshapeR package (45). The DNA shapes consist of three types of DNA structural orientations:

1. *Inter-base pair features*: shift, slide, rise, tilt, roll, helix twist (HelT).
2. *Intra-base pair features*: shear, stretch, stagger, buckle, propeller twist (ProT), opening.
3. Electrostatic potential and minor groove width (MGW).

**Figure 3.**
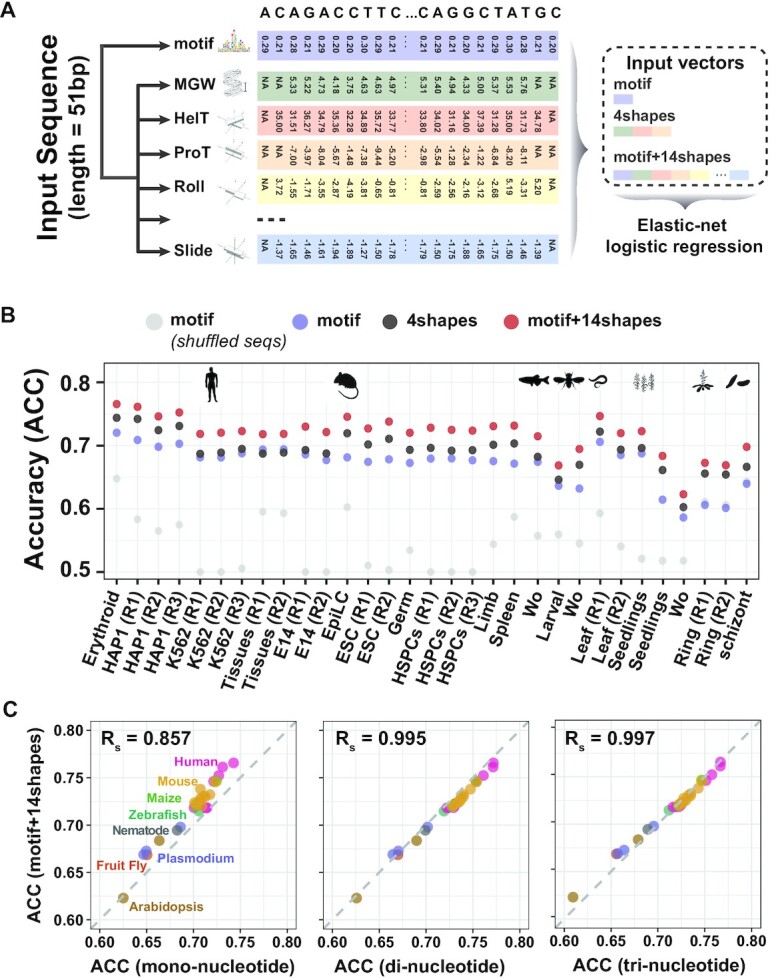
Tn5 preferences for DNA motif and shape are encoded in nucleotide dependence. (**A**) Schematic showing the design of input vectors for machine learning. MGW, minor groove width; HelT, helix twist; ProT, propeller twist. (**B**) Model performance. Models were trained using three types of vectors illustrated in (A). Additionally, the ‘motif (*shuffled seqs*)’ was trained as a negative control to exclude any intrinsic effect from the machine learning algorithm. Model performance was measured using accuracy (ACC). The species is indicated by icons (88). Wo, whole organism. (**C**) Comparison of the information stored in mononucleotide, dinucleotides and trinucleotides with motif+14shapes. For the same dataset used in (B), mononucleotide, dinucleotide and trinucleotide information was calculated as input vectors for training, and the model accuracy was compared with the motif+14shapes model. Each dot represents a sample and is colored according to the corresponding species. *R*_s_ indicates Spearman’s correlation.

The R package DNAshapeR uses sliding pentamer windows obtained through all-atom Monte Carlo simulations to calculate each DNA shape separately. The superiority and robustness of this method were systematically compared (46), which closely correlated with experimental data. The *getShape()* function in the R environment calculates DNA shapes for input fasta files with default parameters (Figure 3; Supplementary Figure S4).

#### Quantitative assessment of effect of DNA shape and motif

We constructed a machine learning framework to assess the quantitative effect of DNA shape and DNA motif on Tn5 insertion preference. Specifically, the elastic-net logistic regression implemented by R package glmnet (47) was used to dissect the relationship between DNA shape, DNA motif and Tn5 insertion event. Three types of vectors (Figure 3A) were used as input:

1. *motif vector:* To fit the width of input sequences (width = 51), we searched for Tn5 motifs for each species using a 51-bp window around Tn5 insertion sites via MEME. The PWM score at each nucleotide around Tn5 insertion sites was calculated for each input sequence.
2. *DNA shape vectors:* DNA shape values were calculated using the DNA sequence around Tn5 insertion sites by the *encodeSeqShape()* function in R package DNAshapeR.
3. *motif + DNA shape vectors:* a combination of the motif vector and DNA shape vectors.

The definition of accuracy is (TP + TN)/(TP + TN + FP +FN), where TP stands for true positive, TN stands for true negative, FP stands for false positive and FN stands for false negative. To reduce the computational cost, we randomly selected 20 000 Tn5 insertion sites and 20 000 random sites across the genome as controls. Because DNA shape and DNA motif have different scales of units, to directly compare their importance, we standardized the input vectors to the [0, 1] range before training. To minimize the overfitting effect on a specific dataset, we used 10-fold cross-validation methods to train the model. To validate the true effect of DNA shape, we shuffled the original pentamer table in ‘TableCompiler.cpp’ three times to break down the original relationships of DNA shapes values, and then we trained the model using *shuffled* 14shapes and true 14shapes for comparison (Supplementary Figure S4B).

### Effect of DNA methylation on Tn5 insertion

#### WGBS data processing

DNA methylation data measured by whole-genome bisulfite sequencing (WGBS) for mouse ESCs and germ cells were downloaded from (48,49). After the general preprocessing procedure described earlier, Bismark version 0.22.3 (50) was used for downstream processing. Specifically, *bismark* and *deduplicate_bismark* subcommands with default parameters were used to map reads to the reference genome and remove duplicated reads, and then *bismark_methylation_extractor* with parameters ‘--no_overlap --ignore 10 --ignore_r2 10 --cytosine_report’ was used to extract the DNA methylation percentage at all cytosine positions. The final bedgraph format files, where the fourth column stands for the percentage of methylation at each cytosine position, were used for downstream analysis.

#### ‘Context-dependent approach’ for dissecting DNA methylation effect

The mouse genome was chopped into tiling 9-bp bins (9mers) using the bedtools *makewindows* command. The DNA methylation level within each bin was calculated by the bedtools *intersect* command. For two cell types, ESCs and germ cells, the methylation level within each bin was compared and classified into one of the following four groups:

1. ESC-only: Methylation level >0 in ESC cell, while = 0 in germ cell.
2. Both: Methylation level >0 in both ESC cell and germ cell.
3. None: Methylation level = 0 in both ESC cell and germ cell.
4. Germ-only: Methylation level = 0 in ESC cell, while >0 in germ cell.

**Figure 4.**
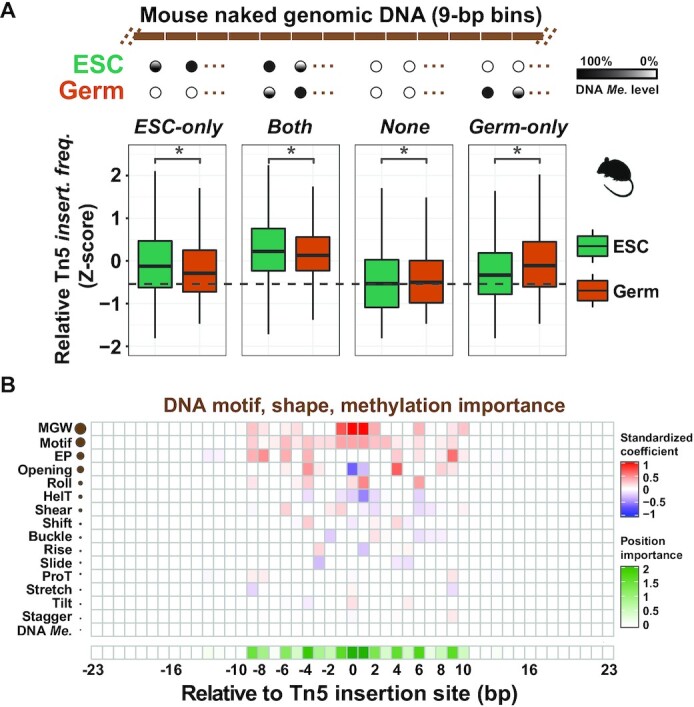
DNA methylation plays a minor role in Tn5 insertion site preference. (**A**) Top panel: Schematic showing the context-dependent approach. Each 9mer along the mouse genome was classified into one of four groups (ESC-only, Both, None, Germ-only), based on the DNA methylation level in mouse ESCs (49) and mouse germ cells (48). For simplification, two 9mers for each group are shown. To maintain the same sequence across four groups, only 9mers that occurred at least once in all four groups (*N* = 165 185) were analyzed (see the ‘Materials and Methods’ section). Bottom panel: Tn5 insertion sites in ESCs (87) and germ cells (48) were mapped to corresponding 9mers. To directly compare the Tn5 insertion frequency (*insert. freq*.) in two cell types, all 9mers in four groups in each cell type were scaled by the *Z*-normalization. (**B**) The relative importance of DNA motif, DNA shape and DNA methylation surrounding Tn5 insertion sites. The contributions of these features are represented by the coefficients returned by the motif+14shapes+DNAme model. The red color in the heatmap indicates a positive role in Tn5 insertion; the blue color indicates a negative role in Tn5 insertion. Each row in the heatmap represents a DNA motif, DNA shape or DNA methylation parameter.

After classification, the Tn5 insertion sites were mapped into corresponding bins. Because each 9mer context will occur many times within each group, for example ‘AAAAAAAAA’ will occur *N* times within the ESC-only groups, we averaged the Tn5 insertion frequency in *N* ‘AAAAAAAAA’ 9mers using bedtools *groupby* command. Following this, we averaged Tn5 insertion frequency at each unique 9mer in each group, for each cell type. To ensure the DNA contexts were the same among all four groups and enable direct comparisons of the DNA methylation effect, we kept 9mers whose sequence occurs in all four groups for downstream analysis. For example, if ‘AAAAAAAAA’ does not occur in any of four groups, we removed this context. After filtering, we got 165 185 ‘shared 9mers’ among all four groups. Because the Tn5 insertion data of ESC and germ cells are from different sources, to directly compare the Tn5 insertion frequency in each 9mer across cell types, we calculated the *Z*-score among all 9mers for each cell type (Figure 4A).

#### Representations of the distribution of Tn5 insertion frequency using boxplot

Boxplot was used for the context-dependent approach throughout the main text to depict the data distribution using the *geom_boxplot()* function in R package ggplot2 (https://ggplot2.tidyverse.org). Before plotting, the top 1% and bottom 1% of outliers were removed to relieve severe data skewness. The line in the middle of a boxplot stands for the median value or the 50th percentile. The lower and upper hinges in the boxplot correspond to the first and third quartiles (the 25th and 75th percentiles). The upper whisker extends from the hinge to the largest value no further than 1.5 × IQR from the hinge (where IQR is the interquartile range or distance between the first and third quartiles). The lower whisker extends from the hinge to the smallest value at most 1.5 × IQR of the hinge. Data beyond the end of the whiskers were called as outliers and are not shown.

### MNase-seq data processing

MNase-seq data for mouse ESCs were downloaded from (51). After the preprocessing procedure (described earlier), raw reads were mapped to the mouse reference genome using Bowtie2, with default parameters. Duplicates were removed using Picard, and bam files were converted to BigWig and bedgraph signal files using deeptools *bamCoverage* at 1-bp resolution. The MNase-seq signals in each 9mer were mapped using bedtools *intersect*.

### ChIP-seq and total RNA-seq data processing

H3K4me1(ENCSR000CGN), H3K4me3 (ENCSR000CGO), H3K27ac (ENCSR000CGQ) and RNA polymerase II (ENCSR000CCC) ChIP-seq as well as total RNA-seq (ENCSR000CWC) data for mouse ESCs were downloaded from ENCODE (31). After the preprocessing procedure (described earlier), raw RNA-seq reads were mapped to the mm10 reference genome using STAR version 2.6.0 (52), with the parameter ‘--quantMode GeneCounts’ used to quantify gene expression using a reference genome annotation file. Afterward, we converted raw counts to transcripts per million using a custom script (code available at https://github.com/YenLab/Tn5InsertPrefer/blob/main/StandaloneScripts/Raw2TPM.R). Raw ChIP-seq reads were mapped to the mm10 reference genome using Bowtie2 with default parameters. ChIP-seq duplicates were removed using Picard. The RNA-seq and ChIP-seq bam files were converted to BigWig format for visualization using deeptools *bamCoverage* at 10-bp resolution.

### Measuring Tn5 insertion preference effect on peak calling analysis

To correct for Tn5 sequence preference (collectively, the motif and shape preferences), we leveraged SeqOutbias version 1.3.0 (53), using a *k*-mer-based dependency matrix to model the Tn5 preference. Based on our observation that a total 19-bp range can affect Tn5 insertion (Figures 2A and 4B), we specifically set *k* = 19 for Tn5 preference correction. We fed a mapped bam file into the SeqOutbias and specified the parameter ‘--no-scale’ to get uncorrected single-base Tn5 insertion signals or the parameter ‘--kmer-mask’ to get corrected signals. The uncorrected and corrected Tn5 insertion signals were used by MACS2 version 2.2.5 (54) for peak calling analysis, with the parameter ‘--broad --format BED --broad-cutoff 0.01 --nomodel --max-gap 100 --shift -100 --extsize 200’ used. The uncorrected and corrected specific peaks were defined by nonoverlapping peaks between uncorrected and corrected peak sets, and any peak sharing at least 1 bp was assigned as a shared peak using the bedtools *intersect* command.

To investigate the enrichment of TFs in peaks, we downloaded all available TF ChIP-seq narrow peaks for mouse ESCs from Cistrome Data Browser (55,56). These 856 ChIP-seq datasets contain 123 TFs (Supplementary Table S3). We used giggle index version 0.6.3 (57) to build a reference for these peaks. We used the giggle *search* command to query the peak set against the reference TF locations for each uncorrected-shared, uncorrected-specific, corrected-shared and corrected-specific peak set. The GC content and peak length-matched control peaks were generated using a custom script (code available at https://github.com/YenLab/Tn5InsertPrefer/blob/main/StandaloneScripts/Negative_sequence_matched_length).

## RESULTS

### Tn5 insertion is not uniformly random in naked genomic DNA

To comprehensively understand the insertion characteristics of Tn5, we first determined whether there are signatures that influence its insertion preference in the absence of chromatin. To this end, we examined a number of publicly available and newly generated Tn5 tagmentation datasets from 20 cell types across eight model organisms (detailed information for each sample is listed in Supplementary Table S2). For the newly generated samples, chromatin was treated with protease to remove chromatin-bound proteins, followed by RNase A treatment to remove single-stranded RNAs. This naked genomic DNA was then treated with Tn5 for tagmentation and adapter ligation, followed by paired-end sequencing. Sequencing reads were mapped to the corresponding reference genome to obtain Tn5 insertion sites (Figure 1, top panel; see the ‘Materials and Methods’ section). As DNA fragment size distribution is a good index to check the genome architecture (5), we restored the fragment size information from paired-end reads and plotted their frequency distribution (Supplementary Figure S1A). We observed a unimodal fragment size distribution in naked genomic DNA as opposed to the periodic nucleosome pattern seen in corresponding chromatin contexts, indicating these samples are indeed naked genomic DNA.

Although the datasets used in our analysis were generated using three sources of Tn5 transposases [Nextera from Illumina, TruePrep from Vazyme and in-house purification (1)], we do not think this will impact our results for three reasons. First, most available Tn5 transposases have been modified following the same guidelines (58). Second, independent work has demonstrated that different Tn5 transposases have similar motifs that are consistent with the earliest description of the wild-type Tn5 motif (10,11,15,17,59). Third, a recent study compared these three Tn5 transposases for constructing RNA-seq libraries and found consistent tagmentation efficiency and gene quantification (60).

Previous evaluations of Tn5 insertion distribution generally relied on Integrative Genomics Viewer (IGV) for visualization. Using IGV, we observed that the Tn5 insertion sites in naked genomic DNA are more dispersed relative to the sharp profile arising from Tn5 tagmentation in chromatin (Supplementary Figure S1B). However, these types of visualizations lack a quantitative measurement. To assess whether Tn5 insertion along naked genomic DNA is a random process, we first focused on the mouse naked genomic DNA to measure Tn5 insertion distribution across 32 genomic features linked with multiple biological functions (Figure 1; see the ‘Materials and Methods’ section). To measure whether Tn5 prefers specific genomic features, we calculated the expected (*E*) Tn5 insertion frequency in each mappable genomic feature for each cell type by assuming a uniformly random distribution. We then compared it with the observed (*O*) Tn5 insertion frequency, using the chi-square test for goodness of fit. The logarithm-transformed *O*/*E* ratio indicates Tn5 preference in a specific genomic feature (Figure 1). Surprisingly, we observed that Tn5 insertions were significantly biased toward/against specific genomic features in the naked genomic DNA of seven cell types. In general, Tn5 exhibited a preference for genic regions (introns, exons and transcription termination sites) as compared to intergenic regions. Strikingly, even in naked genomic DNA, which lacks chromatin-bound proteins such as nucleosomes, Tn5 prefers ELSs (defined by the ENCODE3 project) (7). In contrast, most repetitive regions (LINE, LTR and low-complexity regions) were depleted of Tn5 insertion sites in naked genomic DNA, except for simple repeats, satellites and rRNA regions, which were enriched or depleted of Tn5 insertions depending on the cell type. This observation indicates that the depleted Tn5 insertions were not caused by low DNA extraction efficiency in these heterochromatin regions. Even in naked genomic DNA, we found that Tn5 insertion patterns near TSSs are cell type specific, a signature that could be used to separate stem cells from terminally differentiated cells. A similar Tn5 preference pattern was also observed in human naked genomic DNA (Supplementary Figure S2). These patterns were reproducible among biological replicates and conserved across mouse and human, suggesting that Tn5 has a significant and specific insertion preference, which we sought to investigate further.

### DNA motif signature is insufficient to explain Tn5 insertion preference

DNA-binding protein (DBP) specificity is governed by interactions with either specific nucleotide content (i.e. percentage of AT/GC) or nucleotide composition (i.e. DNA motif) via the DNA-binding domain (61,62). The DNA-binding domain of Tn5 is well resolved (63,64); nevertheless, its nucleotide specificity remains ambiguous.

There are conflicting reports about whether Tn5 prefers AT- or GC-rich regions (17–21); we therefore first tested whether the basis for the insertion preference we observed in genomic features correlates with underlying nucleotide content. To this end, we chose the well-annotated mouse and human naked genomic DNA and compared the relation between Tn5 insertion frequency and nucleotide content in each genomic feature (Figure 1A; Supplementary Figure S2). We did not observe a consistent preference toward AT- or GC-rich nucleotide content in either the mouse or human naked genomic DNA. For example, Tn5 insertions were depleted in both AT-rich (91% AT content) and GC-rich (89% GC content) low-complexity regions of mouse stem cells (Figure 1). To more closely inspect the relation between nucleotide content and Tn5 preference, we analyzed a published hexamer-based evaluation of Tn5 insertion propensity in human naked genomic DNA (24). We ranked and grouped all hexamers based on relative Tn5 insertion frequency and AT content, but we did not find a consistent correlation between AT content and Tn5 insertion frequency across all hexamers (Supplementary Figure S3A). These two results jointly suggest that AT or GC content is not a determinant for Tn5 insertion preference, regardless of species.

We next asked whether Tn5 prefers a particular nucleotide composition (i.e. DNA motif). Searching for a Tn5 motif within a 19-bp window flanking 500 000 randomly selected Tn5 insertion sites using MEME (42) in eight species revealed only one statistically significant motif for each species (Figure 2A; see the ‘Materials and Methods’ section). These Tn5 motifs were consistent with previous reports that a G/C pair occurs at the edge of the 9-bp core motif (14,15,65), except for the motif in *Plasmodium*, a difference that might be driven by its extremely AT-rich genome (81%). We then evaluated the motif contribution for Tn5 insertion by comparing FIMO (43) predicted motif sites with Tn5 insertion sites. We reasoned that if a motif is a strong determinant for Tn5 insertion, most Tn5 insertion sites should fall inside motif sites (which we denoted as ‘inside motif’), and motif sites should largely be engaged for Tn5 insertion (denoted as ‘Used’). However, we found that only 16–29% of the Tn5 insertion sites fall inside motif sites across the eight species we examined (Figure 2B). Because Tn5 will remain at insertion sites, it might obstruct access of another Tn5 at a nearby site (19), in which case saturated motif sites could lead Tn5 to insert into unpreferred sites. We therefore sought to determine whether the Tn5 insertions outside motifs were due to a lack of preferred motifs. When we examined the motif usage along naked genomic DNA, we found that 34–94% of motif sites were used by Tn5 (Figure 2C), depending on the genome size of the species. On average, 39% of motif sites were used in human, mouse, zebrafish and maize, whose average genome size is around 2.4 billion bp, whereas an average of 80% of motif sites were used in fruit fly, nematode, *Arabidopsis* and *Plasmodium*, whose average genome size is around 96 million bp. One possible explanation is that the standard amount of Tn5 used in most tagmentation protocols may be oversaturated for species with smaller genome sizes, and in these cases, Tn5 will insert at weaker motifs.

Using the same motif search strategy for Tn5 insertion sites in chromatin, we identified similar but more GC-rich motifs (Supplementary Figure S3B), which indicates that the DNA motif still contributes to Tn5 insertion preference in a complex chromatin environment. As many Tn5 insertions occur within accessible promoters that overlap with many CpG islands (51), the GC-rich motif might be driven by these GC-rich CpG islands. Nevertheless, these data show that the motif preference is insufficient to fully explain Tn5 insertion specificity.

### DNA shapes contribute cooperatively with DNA motif to affect Tn5 insertion

DNA shapes describe the spatial orientation of inter- or intra-base pairs and have been extensively reported to affect the binding affinity of DBPs through indirect interactions (23,66–68). We first qualitatively measured the effect of the most studied DNA shapes: MGW, HelT, ProT and roll (46) on Tn5 insertion. Because DNA shape might impact a larger window of sequence, we computed DNA shape values within a 51-bp window flanking Tn5 insertion sites or motif sites classified in Figure 2 using DNAshapeR (45) (Supplementary Figure S4A). We found that among these four shapes, motifs that have no Tn5 insertion (motif unused) behave significantly different from sites where Tn5 can insert (motif used, insert outside, insert inside), regardless of the existence of a Tn5 motif, which suggests that DNA shape has an independent role in regulating Tn5 insertion. Generally, Tn5 prefers wider MGW, larger ProT, bigger roll, and smaller HelT, which collectively indicate that Tn5 prefers flexible DNA structures. This may explain why Tn5 shows a preference in naked genomic DNA for ELS regions, as enhancers tend to have larger ProT (69) and their propensity to form chromatin loops suggests their flexibility.

To quantitatively assess the role of DNA shape and DNA motif in Tn5 insertion, we conducted an elastic-net logistic regression framework (47) to hierarchically measure the contribution of DNA motif and 14 types of DNA shapes (70) (Figure 3A; see the ‘Materials and Methods’ section). Briefly, for each naked genomic DNA sample, we randomly chose 20 000 Tn5 insertion sites and 20 000 genomic sites; for each site, the motif and DNA shape values within a 51-bp window were combined into three types of vectors: motif, 4shapes and motif+14shapes. The information encoded in each vector was used to classify Tn5 insertion sites from random genomic sites, and we used the accuracy metric to evaluate the model performance (Figure 3B). We found that the model accuracy ranged from 0.58 to 0.72 among all tested 31 samples across eight species when only the motif vector was fed into the model (motif model). However, the accuracy diminished when trained on shuffled DNA sequence, suggesting that the DNA motif indeed helps Tn5 recognize target sites (Figure 3B). In addition, when trained using shape-containing vectors, the model accuracy increased, further suggesting that DNA shape impacts the Tn5 insertion process. Interestingly, the DNA shape effect can be independent of (4shapes model) or work cooperatively with DNA motif (motif+14shapes model) (Figure 3C). A common issue in machine learning is that additional vectors might increase the model performance simply because more data were involved, even if they contain meaningless information. To rule out this possibility, we shuffled the DNA shape values to break down the original relations for training. We found that the shuffled DNA shapes lost model accuracy compared to true DNA shapes (Supplementary Figure S4B), which confirmed that DNA shapes indeed encode information relevant for Tn5 insertion.

Although Tn5 insertion landscape in a chromatin context was mainly determined by local chromatin architecture, like nucleosomes, we sought to investigate whether DNA shapes affect Tn5 insertion in the chromatin environment. For simplicity, we trained two types of vectors using Tn5 insertion sites in chromatin and found that the motif+14shapes model outperformed the motif-only model in all 23 samples across eight species (Supplementary Figure S4C). Thus, even though nucleosomes serve as a major barrier for Tn5 insertion in chromatin, this result suggests that DNA shapes still contribute to Tn5 preference in this context.

### The intrinsic DNA motif and shape preference originates from nucleotide dependence

The cooperative role of DNA motif and shape led us to ask whether this represents a conserved preference for Tn5 insertion across species. To test this, we randomly divided the data in each of 31 naked DNA samples into a training dataset (70%) and a test dataset (30%). For each sample, we trained a motif+14shapes model (similar to that illustrated in Figure 3A) using its training dataset and tested this model on the other 30 samples using their corresponding test datasets for cross-validation. We observed an overall high cross-validation accuracy; for example, the model trained using human samples can accurately perform in mouse. This suggests that the DNA motif and shape are conserved parameters affecting Tn5 insertion across species (Supplementary Figure S4D). In addition, this indicates that our model did not suffer from overfitting during training.

We next sought to trace the origin of their cooperativity, as DNA motif and DNA shape were both calculated based on DNA sequence. The DNA motif, generally represented by PWM, assumes that the nucleotide at each position contributes independently to the overall affinity (61). However, DNA shapes arise from DNA sequence dependence; for example, base-stacking interactions including HelT and roll were stored as dinucleotide dependences (68,71–73). To test whether the effect of DNA shape and motif on Tn5 insertion can be mimicked through nucleotide dependence information from the DNA sequence, we again used 31 samples from Figure 3B. For sites in each sample, we encoded three types of vectors: mononucleotide, dinucleotide and trinucleotide information, surrounding a 51-bp window for each site for training. In addition, the motif+14shapes model (Figure 3B) was used for comparison. As expected, the motif+14shapes achieved overall higher accuracy for all samples than the mononucleotide model, which did not consider nucleotide dependence (Figure 3C). When dinucleotide information, which encodes dependences between adjacent nucleotide positions, was used as the input vector, the model achieved similar accuracy compared with the motif+14shapes model (Spearman’s correlation = 0.995) (Figure 3C). The accuracy further increased slightly with the trinucleotide model (Figure 3C). These results collectively suggest that even dinucleotide dependence can essentially mimic the information encoded in DNA motif and DNA shapes. This finding may explain the superior performance of Tn5 bias correction methods that consider nucleotide dependence information (10,11,53), as they internally correct for DNA shape effects in practice.

Taken together, our data reveal that DNA motif and DNA shape, the latter of which encodes complementary information about DNA sequence dependence, cooperatively affect Tn5 insertion. However, the cell type-specific Tn5 insertion patterns observed in mouse naked genomic DNA (all of which have the same sequence) (Figure 1) suggest that other transcription-associated features beyond DNA sequence could affect Tn5 insertion.

### DNA methylation makes minimal contributions to Tn5 insertion preference

DNA methylation, which can affect the binding affinity of DBPs (23,74,75), was reported not to affect Tn5 insertion in chromatin (22). Given the complexity of chromatin, however, we sought to investigate the impact of DNA methylation on Tn5 insertion in naked genomic DNA where DNA methylation still exists. To eliminate the influence of DNA sequence, either directly or indirectly, on Tn5 insertion, we focused on available DNA methylation datasets from mouse ESCs (49) and germ cells (48).

We adapted a hexamer-based method that was developed to investigate the effect of DNA methylation on DNase I cleavage (23). Briefly, we classified all 9mers along the naked genomic DNA of mouse ESCs and germ cells into one of four groups according to the DNA methylation level in these cell types: ESC-only, Both, None, and Germ-only. We then looked at the distribution of Tn5 insertion sites across these four groups (see the ‘Materials and Methods’ section; Figure 4A). Using this context-dependent approach, we found that in the same region of the genome, if a cell type has higher methylation, there is a corresponding increase in Tn5 insertion relative to the less methylated cell type. When both cell types have methylation (Both) or have no methylation (None), Tn5 insertion frequency is similarly high or low, respectively. These results suggest that DNA methylation promotes Tn5 insertion in naked genomic DNA and might help explain the cell type-specific pattern in Figure 1.

As DNA methylation stoichiometry has been linked with quantitative biological processes (75), we next tested whether such a scenario existed for Tn5 insertion. We first stratified all genome-wide 9mers into 11 levels based on DNA methylation level in mouse ESCs. Then, we mapped Tn5 insertion frequency in naked DNA (Supplementary Figure S5A) and chromatin (Supplementary Figure S5B) in corresponding 9mers. We observed that compared with the unmethylated 9mers, methylated 9mers have higher Tn5 insertion frequency, consistent with our previous results (Supplementary Figure S5A). However, for methylated 9mers (1–10 level), no apparent quantitative correlation between DNA methylation level and Tn5 insertion frequency was observed in naked genomic DNA (Supplementary Figure S5A). In contrast, in the chromatin context, a clear negative trend was observed (Supplementary Figure S5B). DNA methylation has been positively associated with nucleosome occupancy (76), suggesting that this negative trend might reflect obstruction of Tn5 from the DNA by nucleosomes. However, analysis within accessible chromatin regions [defined by MACS2 peak caller (54)] showed a similar negative trend for both naked genomic DNA and chromatin (Supplementary Figure S5C and D), suggesting that other transcription-associated confounding factors still exist in naked genomic DNA that affect Tn5 insertion, such as other types of DNA modification.

Given that epigenomic features (e.g. DNA methylation) can affect Tn5 insertion, we sought to determine the importance of these features compared with intrinsic parameters (DNA motif and shape). To this end, we again leveraged the logistical regression framework in the naked DNA of mouse ESCs, using a combination of DNA methylation, DNA motif and DNA shape as input vectors for training. As these parameters were standardized to the same range before feeding into the model, we could directly compare each parameter’s coefficient as a proxy for their relative importance on Tn5 insertion (Figure 4B). We calculated total parameter importance and position importance by summing absolute coefficients in each row and column. Strikingly, we found that MGW ranked first, even exceeding the total importance of DNA motif, further highlighting the predominant role of DNA shape in biasing Tn5 insertion (Figure 4B). The importance of this feature may explain the 10-bp periodicity in Tn5 tagmented fragment size distribution that we (Supplementary Figure S1A) and others (5,8) have observed. Consistent with previous reports (22), we found that DNA methylation has negligible overall importance compared with DNA motif and shape, suggesting that it plays only a minor role in affecting Tn5 insertion.

### Correcting for Tn5 insertion preference in ATAC-seq data recovers biologically relevant regulatory information

Based on our finding that nucleotide dependence information can faithfully model DNA motif and shape preference, we sought to investigate whether using this strategy for bias correction could benefit general ATAC-seq applications. It is widely thought that bias correction is more critical for single-base resolution footprinting analysis, and several methods have been developed specifically for this purpose (10,11,24,53). As TFs mainly function in CREs (62), their footprint often depends on the accessible regions defined by peak callers. Peak calling has been widely adopted to find enriched Tn5 insertion sites by comparing the signal in candidate peaks with background signals, the results of which are heavily dependent on the local signal intensity (54). Our results suggest that the local signal can be affected by Tn5 preference, and we therefore sought to determine whether correcting for Tn5 preference can improve peak calling.

**Figure 5.**
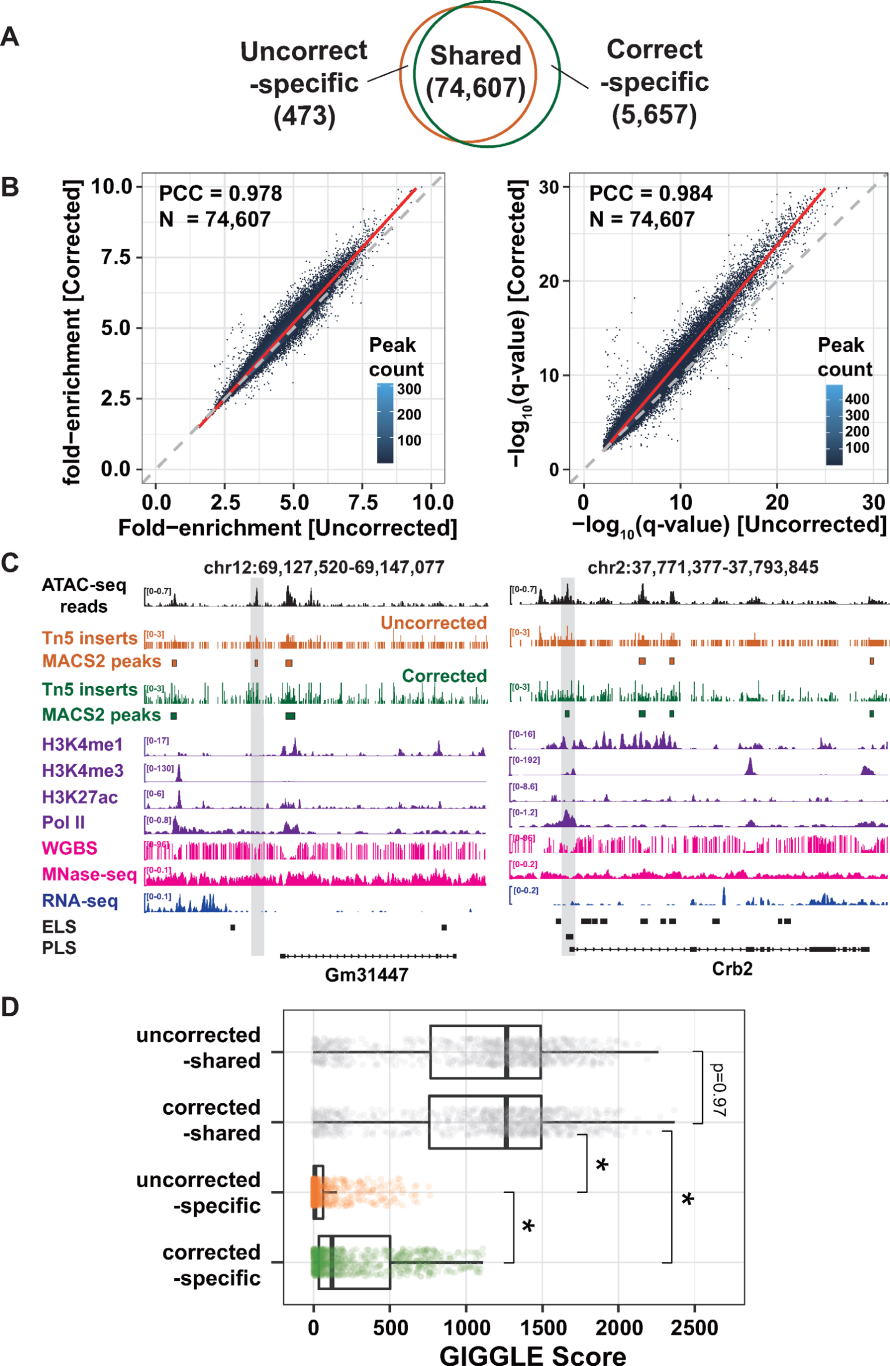
Correcting for Tn5 insertion preference recovers transcriptional regulatory information. (**A**) Venn diagram showing the overlap between uncorrected and corrected peak numbers. (**B**) Comparison of peak calling performance for shared peaks. Left panel: Density plot representation of the ‘fold enrichment at peak submit’ for pairwise shared peaks. Right panel: The ‘−log_10_(*q*-value)’ for pairwise shared peaks. PCC, Pearson correlation coefficient. (**C**) IGV visualization of the uncorrected and corrected peaks and associated epigenomic landscape. The left panel shows an uncorrected-specific peak, and the right panel shows a corrected-specific peak. The bottom shows the ENCODE CREs and RefSeq gene annotation. (**D**) TF enrichment analysis. Each dot indicates one of the 856 TF ChIP-seq datasets. Significance was assigned with *P* < 0.001 as a threshold (Wilcoxon–Mann test).

To correct for the intrinsic DNA sequence preference of Tn5, we leveraged the seqOutBias algorithm (53), which uses a *k*-mer-based method to consider higher order nucleotide dependence for correction. We corrected the mouse ESC ATAC-seq dataset (used in Supplementary Figure S5B) and fed both the uncorrected and corrected signals into the MACS2 peak caller (54) for peak identification. Under the peak calling threshold *q* < 0.01, we detected 75 017 and 80 041 peaks for uncorrected and corrected ATAC-seq signals, respectively (Figure 5A). Among these peaks, 74 607 were shared between uncorrected and corrected signals; 473 and 5657 peaks were specific to uncorrected and corrected signals, respectively.

Although most peaks were shared, we sought to inspect whether bias correction can still benefit these shared peaks in terms of increased peak detection significance. Given the similar coordinates of these shared peaks along the genome, we checked the peak occupancy and significance using the metric ‘fold enrichment at peak submit’ and ‘−log_10_(*q*-value)’ returned by MACS2 (Figure 5B). Interestingly, we found that peak occupancy increased slightly while peak significance improved substantially, suggesting that correction can help improve the overall confidence of peak calling detection by more clearly distinguishing the peak signals from background signals.

To understand the biological significance of these uncorrected- and corrected-specific peaks, we downloaded publicly available epigenome data for mouse ESCs from ENCODE (31), including the H3K4me1, H3K4me3, H3K27ac, RNA polymerase II (Pol II), WGBS and MNase-seq, which collectively can be used to comprehensively evaluate transcriptional status. RNA-seq data were also used to measure the transcription output (Figure 5C; see the ‘Materials and Methods’ section). By visualizing these signals in IGV, we observed an uncorrected-specific peak upstream of the *Gm31447* gene, which has low H3K4me1, H3K4me3, H3K27ac and Pol II signals, but high WGBS and MNase-seq signals (Figure 5C, left panel). This peak did not overlap with any ENCODE CREs, suggesting that it does not have a regulatory function. In contrast, we observed a corrected-specific peak upstream of the *Crb2* TSS, which was associated with low H3K4me1, H3K27ac, WGBS and MNase-seq signals. However, high H3K4me3 and Pol II signals were observed, which jointly indicate that it might be a promoter element (Figure 5C, right panel). Indeed, this peak overlaps with a PLS defined by ENCODE (7); furthermore, the *Crb2* gene has a strong RNA-seq signal, suggesting that this peak is an active promoter.

We next sought to examine the functional landscape of these peaks on a genome-wide scale. To this end, we downloaded all available 856 TF ChIP-seq datasets from the Cistrome Data Browser (55,56), which contains 123 types of TFs (see Supplementary Table S3). We built reference TF binding sites (TFBSs) for these datasets using the GIGGLE algorithm (57), and then we searched the four peak sets (uncorrected-shared, corrected-shared, uncorrected-specific and corrected-specific) against the reference TFBSs for enrichment analysis (Figure 5D). For each peak set, the GIGGLE score was calculated for each TF dataset to represent a composite of TF enrichment and significance. We observed that the uncorrected-shared and corrected-shared peaks have similar high TF enrichment, indicating high transcription regulatory potential. Interestingly, we found that the uncorrected-specific peaks have low TF enrichment (median GIGGLE score: 8.3), implying these peaks might be false positives that were called due to the Tn5 insertion preference. Strikingly, we found that the corrected-specific peaks have considerable TF enrichment (median GIGGLE score: 120.2), suggesting that correcting for Tn5 preference can recover relevant regulatory information.

Although the GIGGLE score is independent of the size of the peak sets compared (57), we sought to test whether the TF enrichment result was affected by an unequal number of peaks input in our case. When we sampled an equal number of peaks (*n* = 400), we observed consistent results (Supplementary Figure S6A) compared with all peaks used (Figure 5D), suggesting that the TF enrichment analysis is robust. We also searched the genome for a GC content- and peak length-matched region for each peak in our sampled peak sets above (*n* = 400), and then conducted a similar TF enrichment analysis. Again, we did not observe TF enrichment in these negative control regions (Supplementary Figure S6B), suggesting that the observed TF enrichment in our peak sets (Figure 5D) is biologically meaningful.

Collectively, we showed that correcting for the intrinsic Tn5 preference not only improves the overall significance for peak detection but also reduces potential false positives and rescues false-negative peaks. The enriched TFs in these putative false-negative peaks (*n* = 5657) should increase the sensitivity for footprinting analysis, providing a richer view of the regulatory landscape. Our results further suggest a broader opportunity to correct for Tn5 insertion preference in ATAC-seq applications beyond footprinting analysis. To facilitate this, we generated a streamlined pipeline from sequenced raw fastq data to Tn5 preference corrected signals and peaks, which is freely available to the community at https://github.com/YenLab/Tn5InsertPrefer/blob/main/BiasFreeATAC.

## DISCUSSION

In this work, we systematically dissected the Tn5 insertion preference in naked genomic DNA and chromatin across several model organisms and found that Tn5 has a conserved insertion preference. This preference is mainly dictated by intrinsic parameters, including DNA motif and DNA shape, which encode complementary information from nucleotide dependence. We further found that correcting these intrinsic Tn5 insertion preferences improves the performance of peak calling algorithms and recovers more transcriptional regulation information.

Tn5 has been divergently reported to exhibit a preference for GC-rich or AT-rich regions (17,18,20,21). We revisited this issue in the context of naked genomic DNA and found no specific relationship between regional AT/GC content and Tn5 insertion preference (Figure 1; Supplementary Figure S2). Based on our analysis, we propose these inconsistencies may arise from studying different species or chromatin contexts. Leveraging a machine learning framework, we found that DNA motif and DNA shape cooperatively affect Tn5 insertion, while DNA methylation plays a minor role (Figure 4B). This quantitative result suggests that it is likely unnecessary to correct for the effect of DNA methylation in ATAC-seq data analysis pipelines. Nevertheless, we cannot exclude the possibility that the DNA methylation effect on Tn5 insertion could be amplified during DNA methylation-associated sequencing protocols, like BS-tagging (77) and methyl-ATAC-seq (78). The quantitative measurement also revealed that wider MGW is the most influential intrinsic parameter. DNase I was reported to prefer narrower MGW (23), suggesting that different enzymes have unique DNA structure preferences that are not captured with a single DNA motif metric (79). This helps to explain why the DNase bias correction strategy cannot be directly applied for Tn5 and motivated us to specifically model Tn5 preference using the nucleotide dependence information for computational correction.

To date, Tn5 preference correction has been seen as more relevant for analyzing footprinting data and has not been considered for peak calling algorithms because it was generally thought that this type of global analysis would not suffer from Tn5 preference. Nevertheless, it was recently proposed by the MACS2 team that bias correction before peak calling might improve performance (80). Indeed, we found that bias correction recovers many potential false-negative peaks with considerable TF enrichment (Figure 5C and D). We propose correcting peaks prior to later analysis could also improve the overall performance of footprinting algorithms. Specifically, the addition of putative false-negative peaks could increase the sensitivity of footprinting. Another potential benefit is that including these peaks might improve the accuracy of footprinting, as distinguishing bound or unbound TF depends on the total number of peaks as background (10,11). Given these potential advantages, we suggest that analysis pipelines be modified to change the order of actions such that bias correction precedes peak calling and footprinting. Such a change requires no extra computational cost but could broaden the value of the results. We anticipate the pipeline developed here will mitigate the impact of Tn5 insertion bias on a number of applications.

Tn5 has boosted the development of high-throughput sequencing methods for studying biological processes, especially at the single-cell level (Supplementary Table S1). However, current single-cell data are sparse and noisy along the genome, frequently requiring imputation or amplification of signals (81–83). Our systematic exploration of Tn5 insertion preference may help to relieve the skewness of amplified signals stemming from Tn5 insertion preference. Additionally, Tn5 tagmentation patterns reflect the biophysical and biochemical DNA properties along the genome, which can be deduced from information datasets generated with Tn5. We envisage more detailed and comprehensive future explorations of datasets derived from protocols involving Tn5 will lead to a deeper understanding of the genome and transcriptional regulation.

## DATA AVAILABILITY

In-house generated Tn5 insertion data using naked genomic DNA are available at NCBI Gene Expression Omnibus (https://www.ncbi.nlm.nih.gov/geo/) under accession number GSE164997. Custom code used to generate the figures for this paper can be downloaded from the GitHub repository (https://github.com/YenLab/Tn5InsertPrefer).

## Supplementary Material

lqab094_Supplemental_Files

## Notes

Present address: Houyu Zhang, Chinese Institute for Brain Research, Academy for Advanced Interdisciplinary Studies, Peking University, Beijing 100871, China.

Present address: Jianyu Yang, Bioinformatics and Genomics Intercollege Graduate Program, Pennsylvania State University, University Park, PA 16802, USA.
