# Supplementary material for: Comprehensive understanding of Tn5 insertion preference improves transcription regulatory element identification
Source: NAR Genom Bioinform. 2021 Oct 27;3(4):lqab094. doi: 10.1093/nargab/lqab094 (PMC8557372; doi:10.1093/nargab/lqab094)
Supplement: lqab094_Supplemental_Files [file lqab094_supplemental_files.zip › Tn5 bias revised Supplementary manuscript resubmission 2.pdf]

Supplemental Figure 1, related to Figure 1. Properties of Tn5-fragmented sequence data in naked genomic DNA and chromatin

(A) Size distribution of Tn5 segmented DNA fragments in 8 species. Blue lines represent samples from chromatin, and green lines represent samples from naked genomic DNA. The detailed information for each sample is listed in ***Table S2***.

(B) The Integrative Genomics Viewer (IGV) visualization of Tn5 insertion site distribution. The Hox gene cluster that functions in stem cells was selected as an example in naked genomic DNA and chromatin for E14 and HSPCs.

Supplemental Figure 2, related to Figure 1. Tn5 exhibits a preference for specific DNA features in human naked genomic DNA

Distribution of Tn5 insertion sites across genomic features in human naked genomic DNA. Red indicates the enrichment of Tn5 insertion sites; blue indicates depletion. Non-significant values (N.S.) are colored gray, with FDR < 0.001 as a threshold (Chi-square test corrected by false discovery rate [FDR]). Cell types were clustered using the "complete" algorithm. The Tn5 source for each sample is indicated. K562 data was generated in this study; public data for erythroid (1), tissues (2), and HAP1 (3) were used in this analysis. Tissues, samples from mixed tissues; R, biological replicates. Genomic feature abbreviations: dELS or pELS, distal or proximal enhancer like signatures; PLS, promoter like a signature; CTCF only, only CTCF bound regions; H3K4me3-DNase, both H3K4me3 and DNase I hypersensitive peaks occur in these regions; (CTCF), CTCF bound in these regions (4); SINE, short interspersed nuclear elements; LINE, long interspersed nuclear elements; LTR, long terminal repeats; Low Comp., low complexity regions. (5).

Supplemental Figure 3, related to Figure 2. Exploration of the Tn5 insertion sequence preference

(A) Tn5 insertion frequency in hexamer contexts. All 4,096 hexamers from (6) were ranked based on the Tn5 insertion frequency. Left panel: top 500 hexamers with highest Tn5 insertion frequency. Middle panel: intermediate hexamers. Right panel: bottom 500 hexamers with lowest Tn5 insertion frequency. Within each panel, hexamers were grouped based on AT content, and the Tn5 insertion frequency for each group is represented as a boxplot. Significance was assigned with P < 0.001 as a threshold (Wilcoxon–Mann test). N.S., not significant.

(B) Tn5 insertion preference for sequence motif in a chromatin context. Motifs found at Tn5 insertion sites in 8 species were returned by *MEME* (7) using a 19-bp window around the Tn5 insertion site.

Supplemental Figure 4, related to Figure 3. Robustness of machine learning framework for exploring the effect of DNA shape on Tn5 insertion

(A) Effect of DNA shape on Tn5 insertion preference. Four types of DNA shapes were plotted: motif sites containing a Tn5 insertion (motif used), motif sites lacking a Tn5 insertion (motif unused), Tn5 insertion sites that fall inside motifs (insert inside), and Tn5 insertion sites that fall outside motifs (insert outside). Additionally, we generated three negative controls: *shuffled* indicates a sequence from the insert inside group that maintains the same nucleotide content but in random order; *matched* indicates the same %GC composition distribution from a set of genomic background sequences; *random* indicates a sequence randomly selected from the genome. The unit of each DNA shape is MGW (Angstrom), HelT (degree), ProT(degree), Roll (degree). The significance between the "motif used" group and the "motif unused" group was assigned with $P < 0.001$ as a threshold (Wilcoxon–Mann test).

(B) Validation of the DNA shape effect. We trained the model using *shuffled* 14shapes and true 14shapes for comparison. Each dot represents a sample and is colored according to the corresponding species.

(C) Effect of DNA shape on Tn5 insertion in a chromatin context. The logistic regression model was used to classify Tn5 insertion sites from random genomic sites using motif or motif+14shapes as input vectors.

(D) Cross-species validation of model performance. Each row or column stands for a sample. Corresponding colors indicate the species as in Figure 3C.

Supplemental Figure 5, related to Figure 4. Quantification of DNA methylation effect in naked genomic DNA and chromatin

(A) and (B). DNA methylation effect on a genome-wide scale. Genome-wide 9mers in ESCs were quantified into deciles based on the DNA methylation level. "1" indicates all 9mers in this group have 1%-10% DNA methylation level and so on. Tn5 insertion frequencies in naked genomic DNA (A) or chromatin (B) were mapped to corresponding 9mers. To keep the sequence pool the same for all DNA methylation levels, only 9mers that occurred at least once in all deciles were analyzed.

(C) and (D). DNA methylation effect within accessible chromatin regions. Similar to (A) and (B), but with the analysis limited to within accessible chromatin regions called by MACS2 using chromatin data. Tn5 insertion frequencies in naked genomic DNA (C) or chromatin (D) were mapped to corresponding 9mers.

Significance in all four panels was assigned with $P < 0.001$ as a threshold (Wilcoxon–Mann test).

Supplemental Figure 6, related to Figure 5. Transcription factor enrichment analysis using negative controls

(A) Similar to Figure 5D, but using a set of 400 randomly selected peaks in each peak set for comparison.

(B) Similar to Figure 5D, but using GC- and peak length-matched negative controls for each peak set as input.

Significance was assigned using the Wilcoxon–Mann test.

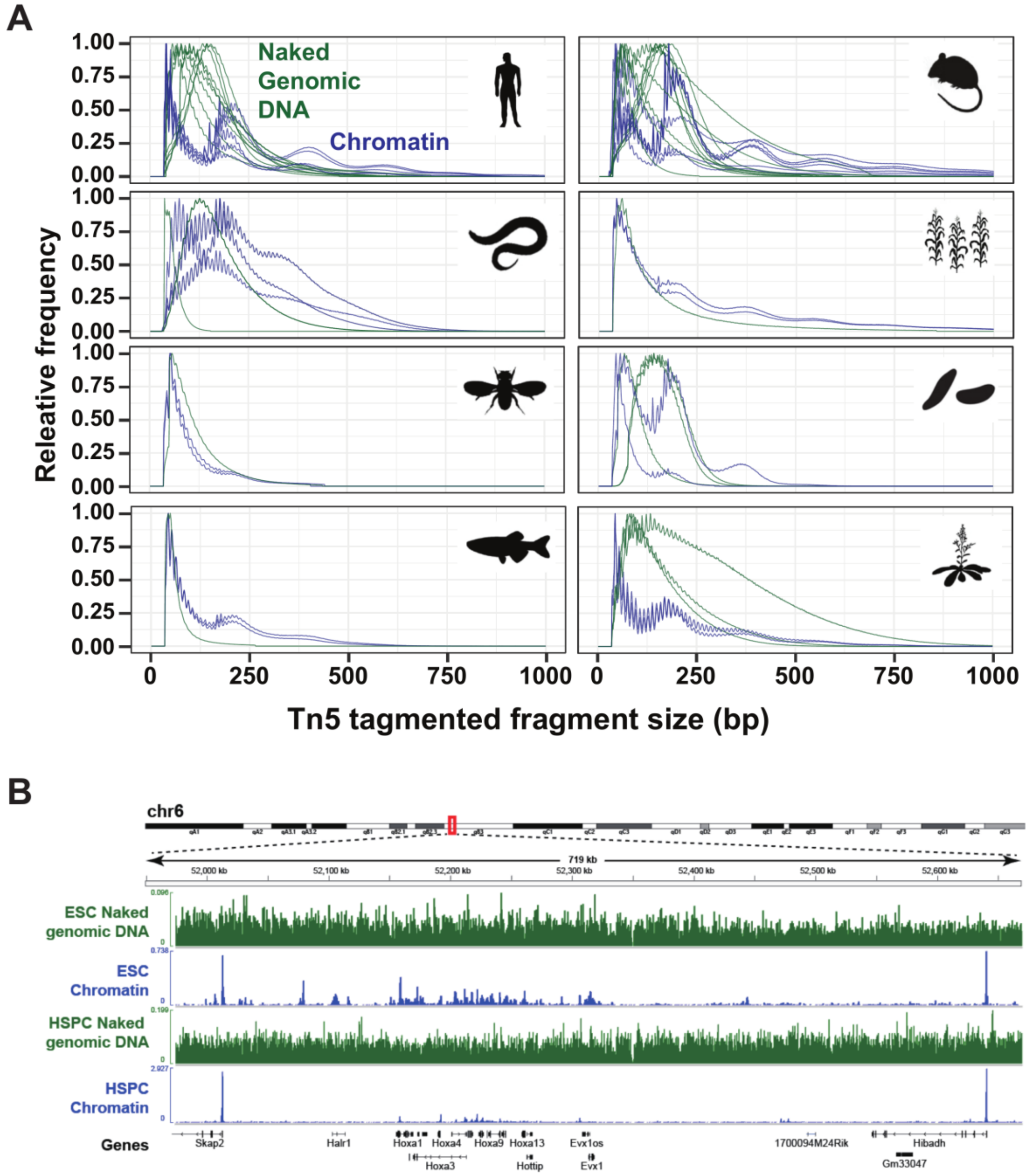
A
Naked
Genomic
DNA
Chromatin
Releative frequency
1.00
0.75
0.50
0.25
0.00
0
250
500
750
1000
Tn5 tagmented fragment size (bp)
B
chr6
719 kb
52,000 kb
52,100 kb
52,200 kb
52,300 kb
52,400 kb
52,500 kb
52,600 kb
ESC Naked genomic DNA
ESC Chromatin
HSPC Naked genomic DNA
HSPC Chromatin
Genes
Skap2
Halr1
Hoxa1
Hoxa4
Hoxa9
Hoxa13
Evx1os
1700094M24Rik
Hibadh
Hoxa3
Hottip
Evx1
Gm33047

Figure S1

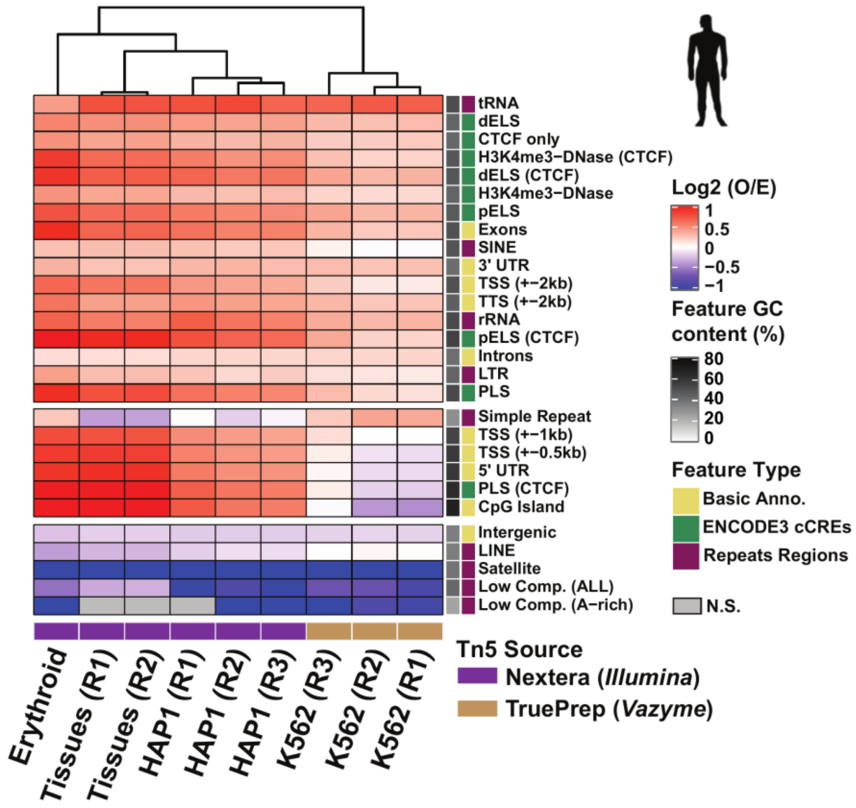
tRNA
dELS
CTCF only
H3K4me3−DNase (CTCF)
dELS (CTCF)
H3K4me3−DNase
pELS
Exons
SINE
3' UTR
TSS (+−2kb)
TTS (+−2kb)
rRNA
pELS (CTCF)
Introns
LTR
PLS
Simple Repeat
TSS (+−1kb)
TSS (+−0.5kb)
5' UTR
PLS (CTCF)
CpG Island
Intergenic
LINE
Satellite
Low Comp. (ALL)
Low Comp. (A−rich)
Log2 (O/E)
1
0.5
0
−0.5
−1
Feature GC content (%)
80
60
40
20
0
Feature Type
Basic Anno.
ENCODE3 cCREs
Repeats Regions
N.S.
Tn5 Source
Nextera (Illumina)
TruePrep (Vazyme)
Erythroid
Tissues (R1)
Tissues (R2)
HAP1 (R1)
HAP1 (R2)
HAP1 (R3)
K562 (R3)
K562 (R2)
K562 (R1)

Figure S2

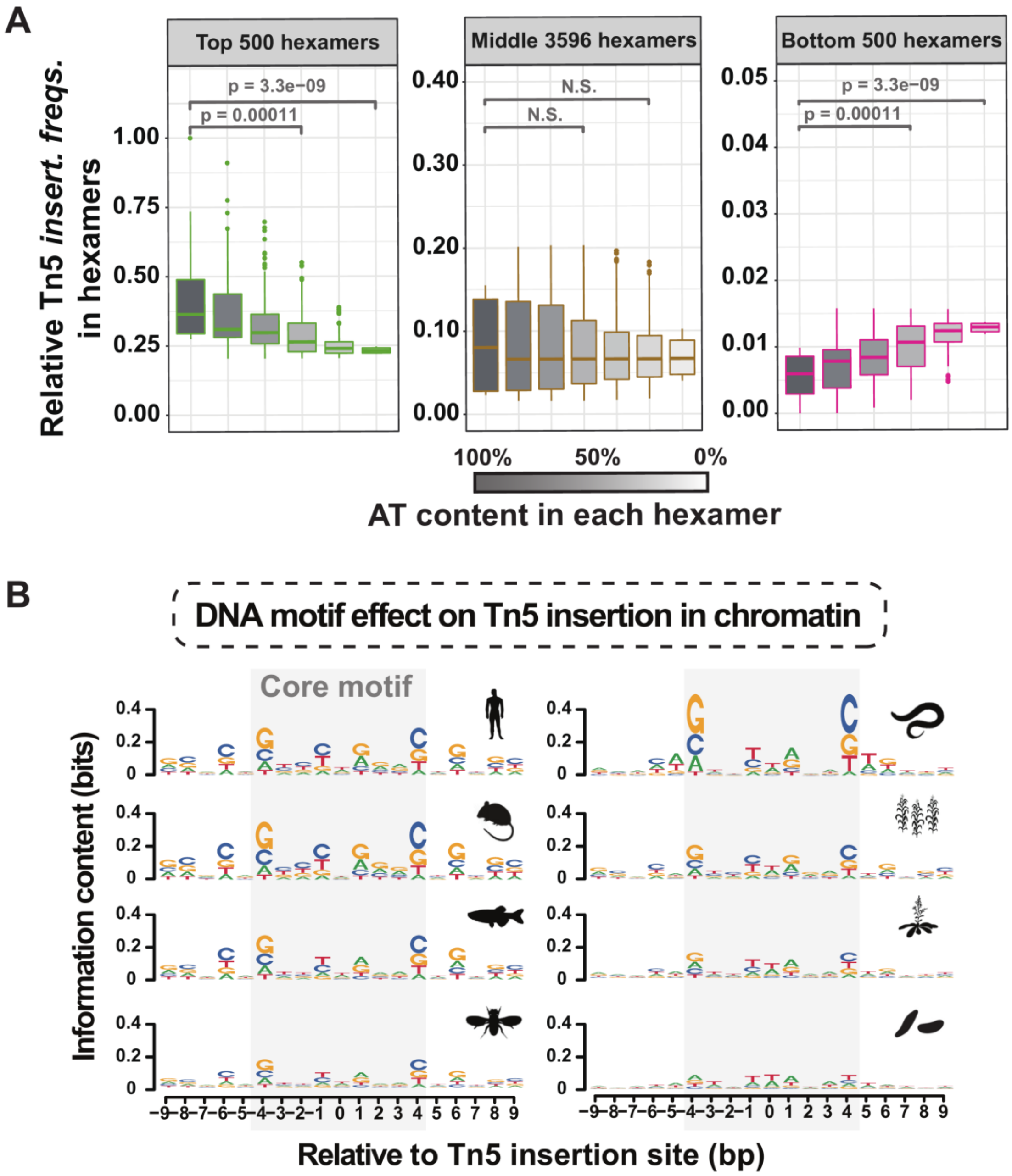
A
Top 500 hexamers
Middle 3596 hexamers
Bottom 500 hexamers
p = 3.3e−09
p = 0.00011
N.S.
N.S.
p = 3.3e−09
p = 0.00011
Relative Tn5 insert. freqs. in hexamers
100%
50%
0%
AT content in each hexamer
B
DNA motif effect on Tn5 insertion in chromatin
Core motif
Information content (bits)
Relative to Tn5 insertion site (bp)

Figure S3

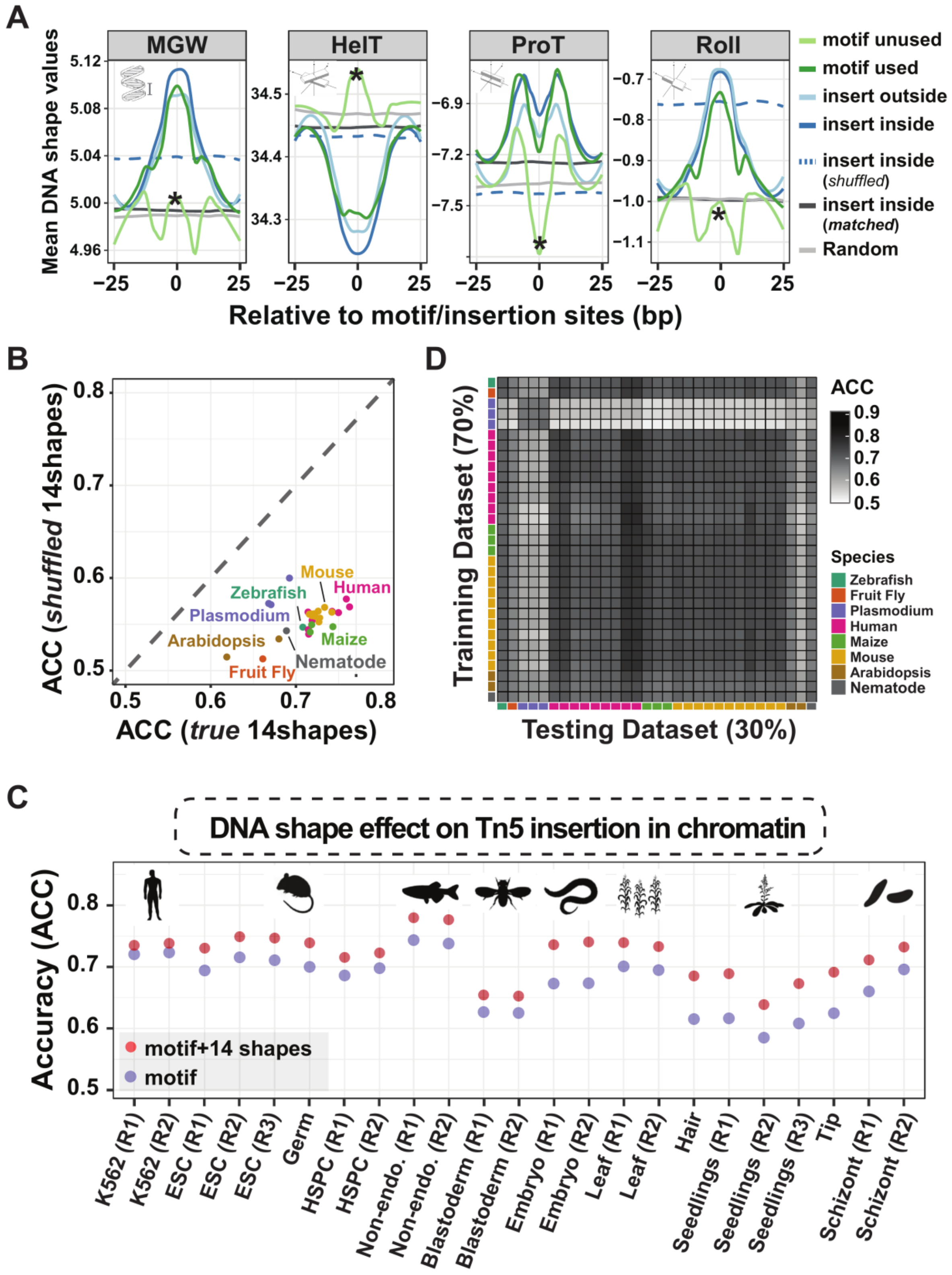
A
MGW
HelT
ProT
Roll
Mean DNA shape values
Relative to motif/insertion sites (bp)
motif unused
motif used
insert outside
insert inside
insert inside (shuffled)
insert inside (matched)
Random
B
ACC (shuffled 14shapes)
ACC (true 14shapes)
Mouse
Human
Zebrafish
Plasmodium
Arabidopsis
Maize
Nematode
Fruit Fly
D
Trainning Dataset (70%)
Testing Dataset (30%)
ACC
Species
Zebrafish
Fruit Fly
Plasmodium
Human
Maize
Mouse
Arabidopsis
Nematode
C
DNA shape effect on Tn5 insertion in chromatin
Accuracy (ACC)
motif+14 shapes
motif
K562 (R1)
K562 (R2)
ESC (R1)
ESC (R2)
ESC (R3)
Germ
HSPC (R1)
HSPC (R2)
Non-endo. (R1)
Non-endo. (R2)
Blastoderm (R1)
Blastoderm (R2)
Embryo (R1)
Embryo (R2)
Leaf (R1)
Leaf (R2)
Hair
Seedlings (R1)
Seedlings (R2)
Seedlings (R3)
Tip
Schizont (R1)
Schizont (R2)

Figure S4

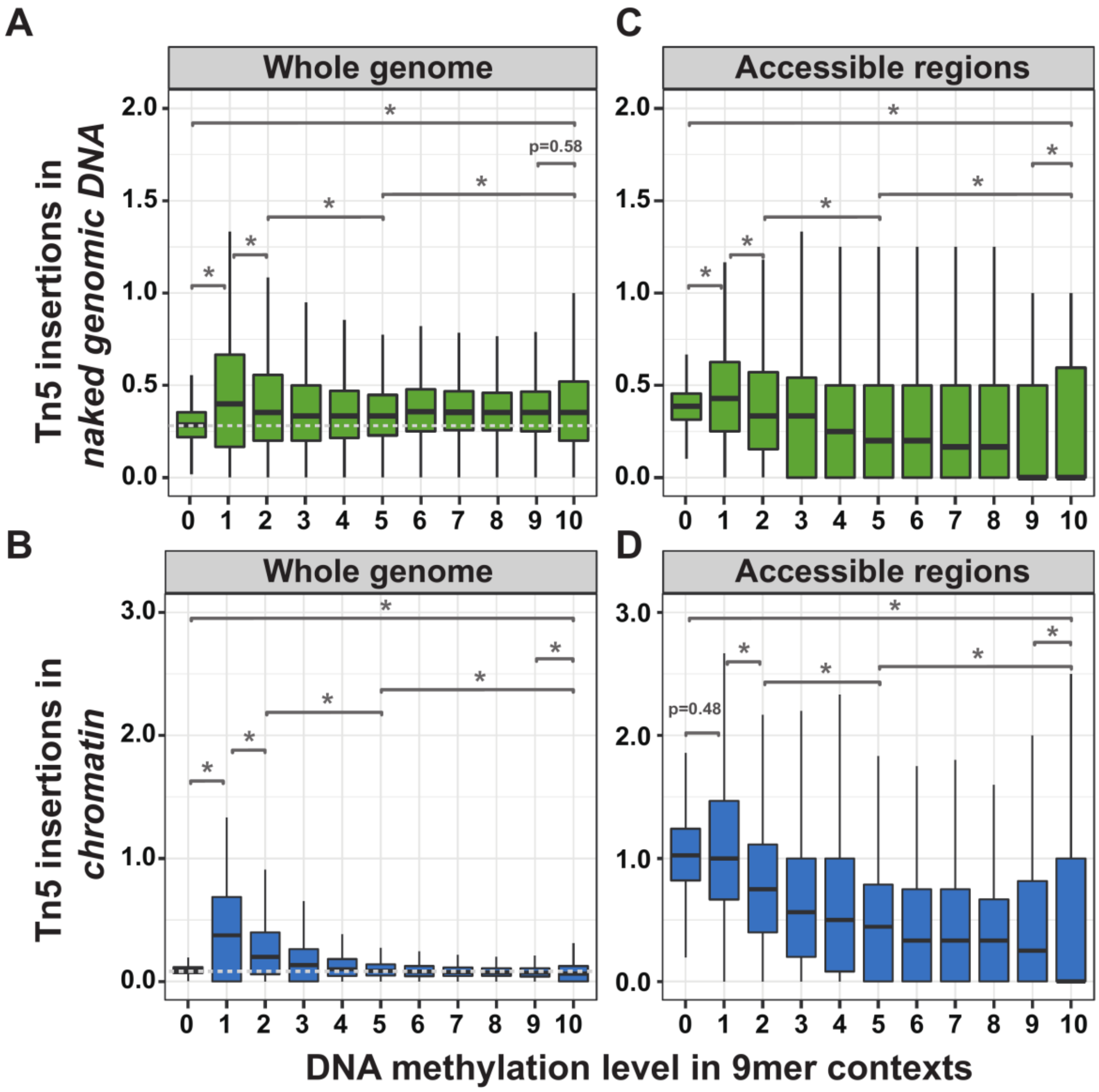
A
B
C
D
Whole genome
Accessible regions
Whole genome
Accessible regions
Tn5 insertions in naked genomic DNA
Tn5 insertions in chromatin
p=0.58
p=0.48
DNA methylation level in 9mer contexts

Figure S5

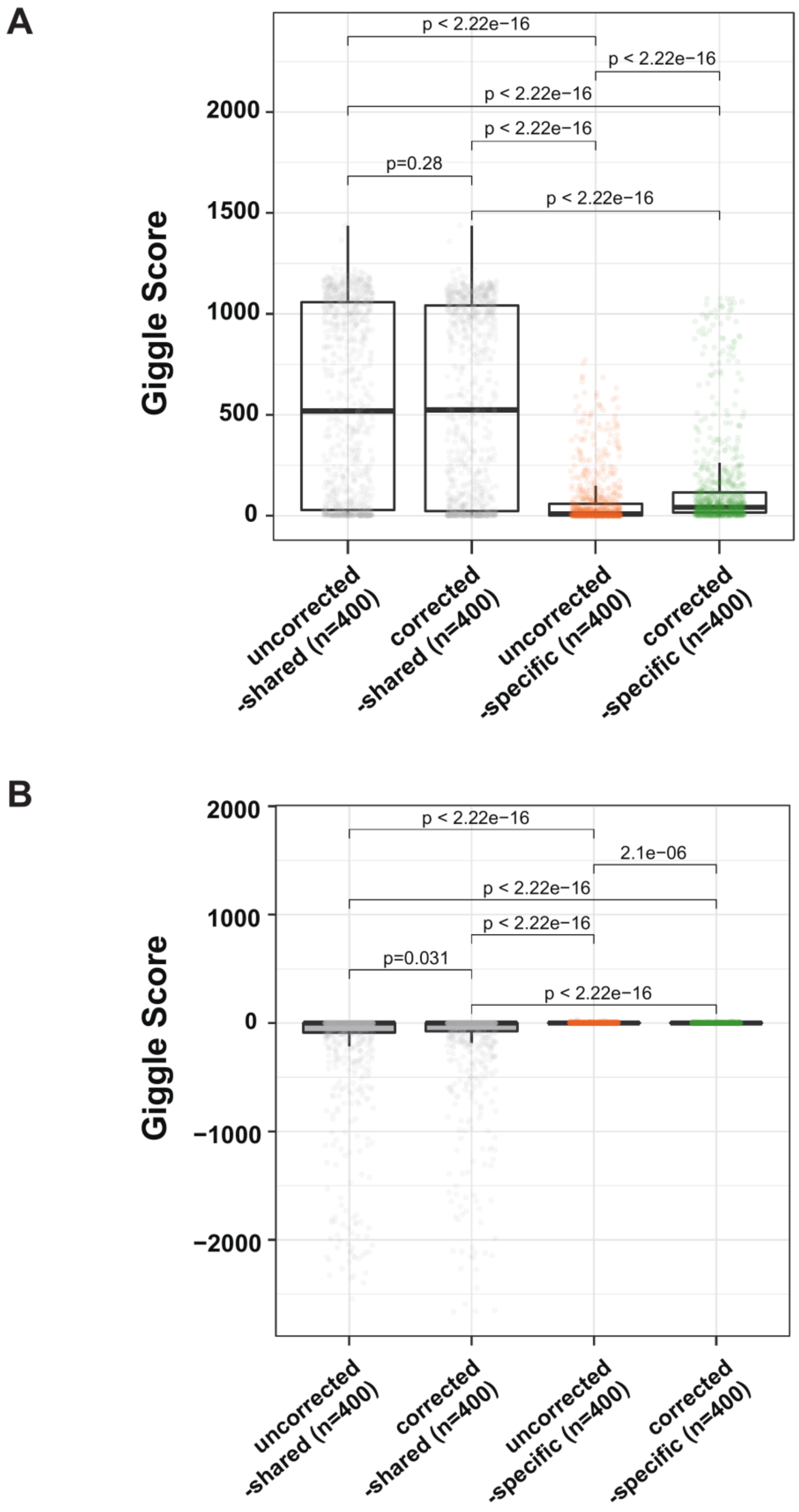
A
p < 2.22e−16
p < 2.22e−16
p < 2.22e−16
p < 2.22e−16
p=0.28
p < 2.22e−16
Giggle Score
2000
1500
1000
500
0
uncorrected -shared (n=400)
corrected -shared (n=400)
uncorrected -specific (n=400)
corrected -specific (n=400)
B
p < 2.22e−16
2.1e−06
p < 2.22e−16
p < 2.22e−16
p=0.031
p < 2.22e−16
Giggle Score
2000
1000
0
−1000
−2000
uncorrected -shared (n=400)
corrected -shared (n=400)
uncorrected -specific (n=400)
corrected -specific (n=400)

Figure S6

Supplemental Table S1. Sequencing protocols using Tn5 for fragmentation

Protocols in **bold** support single-cell inputs.

Protocol name	Assay type	Reference
Shevchenko	cDNA sequencing	(8)
Adey	Sequencing library construction	(9)
Tn5mC-seq	DNA methylation	(10)
Brouilette	RNA-seq	(11)
Tn-RNA-seq	RNA-seq	(12)
ATAC-seq	Chromatin accessibility	(13)
T-WGBS	DNA methylation	(14)
Picelli	Sequencing library construction	(15)
CPT-seq	Whole-genome sequencing	(2)
ChIPmentation	ChIP-seq	(16)
**ATAC-seq**	**Chromatin accessibility**	**(17)**
THS-seq	Chromatin accessibility	(18)
Simul-seq	Chromatin accessibility	(19)
ATAC-see	Chromatin accessibility imaging	(20)
LIANTI	Whole-genome sequencing	(21)
omni-ATAC	Chromatin accessibility	(22)
ChIP-exo	High-resolution ChIP-seq	(23)
**Tn5Prime**	**RNA-seq**	**(24)**
BS-tagging	DNA methylation	(25)
DiP-C	Chromatin architecture	(26)
**snATAC-seq**	**Chromatin accessibility**	**(27)**
sci-CAR	RNA-seq & ATAC-seq	(28)
Trac-looping	Chromatin architecture	(29)
**Plate-based scATAC-seq**	**Chromatin accessibility**	**(30)**
**scNMT-seq**	**DNA methylation & RNA-seq & ATAC-seq in the same cell**	**(31)**
stLFR	long DNA molecules library	(32)

	**molecules**	
**SNARE-seq**	**RNA-seq & ATAC-seq in the same cell**	**(33)**
**ChIL–seq**	**ChIP-seq**	**(34)**
**repli-ATAC-seq**	**Chromatin accessibility**	**(35)**
**Paired-seq**	**RNA-seq & ATAC-seq in the same cell**	**(36)**
**CUT&Tag**	**ChIP-seq**	**(37)**
**ACT-seq**	**ChIP-seq**	**(38)**
**CoBATCH**	**ChIP-seq**	**(39)**
**itChIP-seq**	**ChIP-seq**	**(40)**
**scCAT-seq**	**RNA-seq & ATAC-seq in the same cell**	**(41)**
methyl-ATAC-seq	DNA methylation	(42)
**SHERRY**	**RNA-seq**	**(43)**
**TRACE-seq**	**RNA-seq**	**(44)**
**ASTAR-seq**	**RNA-seq & ATAC-seq in the same cell**	**(45)**
**mtscATAC-seq**	**scATAC-seq for mtDNA**	**(46)**
ATAC-Me	ATAC-seq & DNA methylation	(47)
TagHi-C	Chromatin architecture	(48)
**ASTAR-seq**	**RNA-seq & ATAC-seq in the same cell**	**(45)**
**SHARE-seq**	**RNA-seq & ATAC-seq in the same cell**	**(49)**
Stacc–seq	ChIP-seq	(50)
CUTAC	ChIP-seq & ATAC-seq	(51)
IGS	sequencing and imaging genomes	(52)
**META-CS**	**Whole-genome amplification**	**(53)**
iDAPT-seq	ChIP-seq & ATAC-seq	(54)
**Paired-Tag**	**ChIP-seq & RNA-seq**	**(55)**

Supplemental Table S2. Tn5 insertion data used in this study

Information includes organism, GEO samples number, cell types, genomic context (31 samples from naked genomic DNA and 23 samples from chromatin), and source of Tn5 enzyme for each sample.

Organism	GSM Number	Cell type	Naked genomic DNA /Chromatin	Tn5 enzyme source	Reference
Mus musculus	GSM3426479	HSPC	Chromatin	TruePrep (Vazyme)	(56)
	GSM3426488	HSPC	Chromatin	TruePrep (Vazyme)	(56)
	GSM5024213	HSPC	Naked genomic DNA	TruePrep (Vazyme)	This study
	GSM5024214	HSPC	Naked genomic DNA	TruePrep (Vazyme)	This study
	GSM5024215	HSPC	Naked genomic DNA	TruePrep (Vazyme)	This study
	GSM5024211	E14	Naked genomic DNA	TruePrep (Vazyme)	This study
	GSM5024212	E14	Naked genomic DNA	TruePrep (Vazyme)	This study
	GSM2333647	ESC	Chromatin	Nextera (Illumina)	(57)
	GSM2333648	ESC	Chromatin	Nextera (Illumina)	(57)
	GSM2333649	ESC	Chromatin	Nextera (Illumina)	(57)
	GSM2333650	ESC	Naked genomic DNA	Nextera (Illumina)	(57)
	GSM2333651	ESC	Naked genomic DNA	Nextera (Illumina)	(57)
	GSM1550786	Germ cells	Naked genomic DNA	Nextera (Illumina)	(58)
	GSM1550784	Germ cells	Chromatin	Nextera (Illumina)	(58)
	GSM2981009	EpiLC	Naked genomic DNA	Nextera (Illumina)	(59)

	GSM3439285	Spleen	Naked genomic DNA	In-house	(60)
	GSM4048700	Limb	Naked genomic DNA	Nextera (Illumina)	(61)
Homo sapiens	SRX2918779	Tissues	Naked genomic DNA	EZ-Tn5 (Epicentre, Illumina)	(2)
	SRX660455	Tissues	Naked genomic DNA	EZ-Tn5 (Epicentre, Illumina)	(2)
	GSM3020970	HAP1	Naked genomic DNA	Nextera (Illumina)	(3)
	GSM3020971	HAP1	Naked genomic DNA	Nextera (Illumina)	(3)
	GSM3020972	HAP1	Naked genomic DNA	Nextera (Illumina)	(3)
	GSM2594186	Erythroid	Naked genomic DNA	Nextera (Illumina)	(1)
	ENCLB918NXF	K562	Chromatin	Nextera (Illumina)	(62)
	ENCLB758GEG	K562	Chromatin	Nextera (Illumina)	(62)
	GSM5024216	K562	Naked genomic DNA	TruePrep (Vazyme)	This study
	GSM5024217	K562	Naked genomic DNA	TruePrep (Vazyme)	This study
	GSM5024218	K562	Naked genomic DNA	TruePrep (Vazyme)	This study
Danio rerio	GSM3855036	whole embryo	Naked genomic DNA	Nextera (Illumina)	(63)
	GSM3855021	non-endothelial	Chromatin	Nextera (Illumina)	(63)
	GSM3855022	non-endothelial	Chromatin	Nextera (Illumina)	(63)
Drosophila melanogaster	GSM3323415	larval tissues	Naked genomic DNA	Nextera (Illumina)	(64)
	GSM3323413	blastoderm	Chromatin	Nextera (Illumina)	(64)
	GSM3323414	blastoderm	Chromatin	Nextera (Illumina)	(64)
Caenorhabditis elegans	GSM2385318	whole organisms	Naked genomic DNA	Nextera (Illumina)	(65)
	GSM2385309	early embryo	Chromatin	Nextera (Illumina)	(65)
	GSM2385312	early embryo	Chromatin	Nextera (Illumina)	(65)

Plasmodium falciparum	GSM2789029	ring stage	Naked genomic DNA	Nextera (Illumina)	(66)
	GSM2789030	ring stage	Naked genomic DNA	Nextera (Illumina)	(66)
	GSM2947036	schizont	Naked genomic DNA	Nextera (Illumina)	(67)
	GSM2947026	schizont	Chromatin	Nextera (Illumina)	(67)
	GSM2947027	schizont	Chromatin	Nextera (Illumina)	(67)
Zea mays	GSM3674747	leaf	Naked genomic DNA	Nextera (Illumina)	(68)
	GSM3674748	leaf	Naked genomic DNA	Nextera (Illumina)	(68)
	GSM3401641	Seedlings	Chromatin	Nextera (Illumina)	-
	GSM3401642	Seedlings	Chromatin	Nextera (Illumina)	-
	GSM3401643	Seedlings	Naked genomic DNA	Nextera (Illumina)	-
Arabidopsis thaliana	GSM2704269	Whole organism	Naked genomic DNA	Nextera (Illumina)	(69)
	GSM2704267	hair cell	Chromatin	Nextera (Illumina)	(69)
	GSM2704255	tip cell	Chromatin	Nextera (Illumina)	(69)
	GSM2260241	seedlings	Naked genomic DNA	Nextera (Illumina)	(70)
	GSM2260231	seedlings	Chromatin	Nextera (Illumina)	(70)
	GSM2260232	seedlings	Chromatin	Nextera (Illumina)	(70)
	GSM2260233	seedlings	Chromatin	Nextera (Illumina)	(70)

REFERENCES

1. Schwessinger, R., Suciu, M.C., McGowan, S.J., Telenius, J., Taylor, S., Higgs, D.R. and Hughes, J.R. (2017) Sasquatch: predicting the impact of regulatory SNPs on transcription factor binding from cell- and tissue-specific DNase footprints. *Genome research*, **27**, 1730-1742.
2. Amini, S., Pushkarev, D., Christiansen, L., Kostem, E., Royce, T., Turk, C., Pignatelli, N., Adey, A., Kitzman, J.O., Vijayan, K. *et al.* (2014) Haplotype-resolved whole-genome sequencing by contiguity-preserving transposition and combinatorial indexing. *Nature genetics*, **46**, 1343-1349.
3. Rodriguez-Castaneda, F., Lemma, R.B., Cuervo, I., Bengtsen, M., Moen, L.M., Ledsaak, M., Eskeland, R. and Gabrielsen, O.S. (2018) The SUMO protease SENP1 and the chromatin remodeler CHD3 interact and jointly affect chromatin accessibility and gene expression. *J Biol Chem*, **293**, 15439-15454.
4. Moore, J.E., Purcaro, M.J., Pratt, H.E., Epstein, C.B., Shoresh, N., Adrian, J., Kawli, T., Davis, C.A., Dobin, A., Kaul, R. *et al.* (2020) Expanded encyclopaedias of DNA elements in the human and mouse genomes. *Nature*, **583**, 699-710.
5. Price, A.L., Jones, N.C. and Pevzner, P.A. (2005) De novo identification of repeat families in large genomes. *Bioinformatics*, **21 Suppl 1**, i351-358.
6. Karabacak Calviello, A., Hirsekorn, A., Wurmus, R., Yusuf, D. and Ohler, U. (2019) Reproducible inference of transcription factor footprints in ATAC-seq and DNase-seq datasets using protocol-specific bias modeling. *Genome biology*, **20**, 42.
7. Bailey, T.L., Johnson, J., Grant, C.E. and Noble, W.S. (2015) The MEME Suite. *Nucleic acids research*, **43**, W39-49.
8. Shevchenko, Y., Bouffard, G.G., Butterfield, Y.S.N., Blakesley, R.W., Hartley, J.L., Young, A.C., Marra, M.A., Jones, S.J.M., Touchman, J.W. and Green, E.D. (2002) Systematic sequencing of cDNA clones using the transposon Tn5. *Nucleic acids research*, **30**, 2469-2477.
9. Adey, A., Morrison, H.G., Asan, Xun, X., Kitzman, J.O., Turner, E.H., Stackhouse, B., MacKenzie, A.P., Caruccio, N.C., Zhang, X.Q. *et al.* (2010) Rapid, low-input, low-bias construction of shotgun fragment libraries by high-density in vitro transposition. *Genome biology*, **11**.
10. Adey, A. and Shendure, J. (2012) Ultra-low-input, tagmentation-based whole-genome bisulfite sequencing. *Genome research*, **22**, 1139-1143.
11. Brouilette, S., Kuersten, S., Mein, C., Bozek, M., Terry, A., Dias, K.R., Bhaw-Rosun, L., Shintani, Y., Coppen, S., Ikebe, C. *et al.* (2012) A simple and novel method for RNA-seq library preparation of single cell cDNA analysis by hyperactive Tn5 transposase. *Dev Dyn*, **241**, 1584-1590.
12. Gertz, J., Varley, K.E., Davis, N.S., Baas, B.J., Goryshin, I.Y., Vaidyanathan, R., Kuersten, S. and Myers, R.M. (2012) Transposase mediated construction of RNA-seq libraries. *Genome research*, **22**, 134-141.
13. Buenrostro, J.D., Giresi, P.G., Zaba, L.C., Chang, H.Y. and Greenleaf, W.J. (2013) Transposition of native chromatin for fast and sensitive epigenomic profiling of open chromatin, DNA-binding proteins and nucleosome position. *Nature methods*, **10**, 1213-1218.
14. Wang, Q., Gu, L., Adey, A., Radlwimmer, B., Wang, W., Hovestadt, V., Bahr, M., Wolf, S., Shendure, J., Eils, R. *et al.* (2013) Tagmentation-based whole-genome bisulfite sequencing. *Nature protocols*, **8**, 2022-2032.
15. Picelli, S., Bjorklund, A.K., Reinius, B., Sagasser, S., Winberg, G. and Sandberg, R. (2014) Tn5 transposase and tagmentation procedures for massively scaled sequencing projects. *Genome research*, **24**, 2033-2040.
16. Schmidl, C., Rendeiro, A.F., Sheffield, N.C. and Bock, C. (2015) ChIPmentation: fast, robust, low-input ChIP-seq for histones and transcription factors. *Nature methods*, **12**, 963-965.

17. Buenrostro, J.D., Wu, B., Litzenburger, U.M., Ruff, D., Gonzales, M.L., Snyder, M.P., Chang, H.Y. and Greenleaf, W.J. (2015) Single-cell chromatin accessibility reveals principles of regulatory variation. *Nature*, **523**, 486-490.
18. Sos, B.C., Fung, H.L., Gao, D.R., Osothprarop, T.F., Kia, A., He, M.M. and Zhang, K. (2016) Characterization of chromatin accessibility with a transposome hypersensitive sites sequencing (THS-seq) assay. *Genome biology*, **17**, 20.
19. Reuter, J.A., Spacek, D.V., Pai, R.K. and Snyder, M.P. (2016) Simul-seq: combined DNA and RNA sequencing for whole-genome and transcriptome profiling. *Nature methods*, **13**, 953-958.
20. Chen, X., Shen, Y., Draper, W., Buenrostro, J.D., Litzenburger, U., Cho, S.W., Satpathy, A.T., Carter, A.C., Ghosh, R.P., East-Seletsky, A. *et al.* (2016) ATAC-see reveals the accessible genome by transposase-mediated imaging and sequencing. *Nature methods*, **13**, 1013-1020.
21. Chen, C., Xing, D., Tan, L., Li, H., Zhou, G., Huang, L. and Xie, X.S. (2017) Single-cell whole-genome analyses by Linear Amplification via Transposon Insertion (LIANTI). *Science*, **356**, 189-194.
22. Corces, M.R., Trevino, A.E., Hamilton, E.G., Greenside, P.G., Sinnott-Armstrong, N.A., Vesuna, S., Satpathy, A.T., Rubin, A.J., Montine, K.S., Wu, B. *et al.* (2017) An improved ATAC-seq protocol reduces background and enables interrogation of frozen tissues. *Nature methods*, **14**, 959-962.
23. Rossi, M.J., Lai, W.K.M. and Pugh, B.F. (2018) Simplified ChIP-exo assays. *Nature communications*, **9**, 2842.
24. Cole, C., Byrne, A., Beaudin, A.E., Forsberg, E.C. and Vollmers, C. (2018) Tn5Prime, a Tn5 based 5' capture method for single cell RNA-seq. *Nucleic acids research*, **46**, e62.
25. Suzuki, M., Liao, W., Wos, F., Johnston, A.D., DeGrazia, J., Ishii, J., Bloom, T., Zody, M.C., Germer, S. and Greally, J.M. (2018) Whole-genome bisulfite sequencing with improved accuracy and cost. *Genome research*, **28**, 1364-1371.
26. Tan, L., Xing, D., Chang, C.H., Li, H. and Xie, X.S. (2018) Three-dimensional genome structures of single diploid human cells. *Science*, **361**, 924-928.
27. Preissl, S., Fang, R., Huang, H., Zhao, Y., Raviram, R., Gorkin, D.U., Zhang, Y., Sos, B.C., Afzal, V., Dickel, D.E. *et al.* (2018) snATAC-seq: Single-nucleus analysis of accessible chromatin in developing mouse forebrain reveals cell-type-specific transcriptional regulation. *Nature neuroscience*, **21**, 432-439.
28. Cao, J., Cusanovich, D.A., Ramani, V., Aghamirzaie, D., Pliner, H.A., Hill, A.J., Daza, R.M., McFaline-Figueroa, J.L., Packer, J.S., Christiansen, L. *et al.* (2018) Joint profiling of chromatin accessibility and gene expression in thousands of single cells. *Science*, **361**, 1380.
29. Lai, B., Tang, Q., Jin, W., Hu, G., Wangsa, D., Cui, K., Stanton, B.Z., Ren, G., Ding, Y., Zhao, M. *et al.* (2018) Trac-looping measures genome structure and chromatin accessibility. *Nature methods*, **15**, 741-747.
30. Chen, X., Miragaia, R.J., Natarajan, K.N. and Teichmann, S.A. (2018) A rapid and robust method for single cell chromatin accessibility profiling. *Nature communications*, **9**, 5345.
31. Clark, S.J., Argelaguet, R., Kapourani, C.A., Stubbs, T.M., Lee, H.J., Alda-Catalinas, C., Krueger, F., Sanguinetti, G., Kelsey, G., Marioni, J.C. *et al.* (2018) scNMT-seq enables joint profiling of chromatin accessibility DNA methylation and transcription in single cells. *Nature communications*, **9**, 781.
32. Wang, O., Chin, R., Cheng, X., Wu, M.K.Y., Mao, Q., Tang, J., Sun, Y., Anderson, E., Lam, H.K., Chen, D. *et al.* (2019) Efficient and unique cobarcoding of second-generation sequencing reads from long DNA molecules enabling cost-effective and accurate sequencing, haplotyping, and de novo assembly. *Genome research*, **29**, 798-808.
33. Chen, S., Lake, B.B. and Zhang, K. (2019) High-throughput sequencing of the transcriptome and chromatin accessibility in the same cell. *Nature biotechnology*.
34. Harada, A., Maehara, K., Handa, T., Arimura, Y., Nogami, J., Hayashi-Takanaka, Y., Shirahige, K., Kurumizaka, H., Kimura, H. and Ohkawa, Y. (2019) A chromatin integration

labelling method enables epigenomic profiling with lower input. *Nature cell biology*, **21**, 287-296.
35. Stewart-Morgan, K.R., Reverón-Gómez, N. and Groth, A. (2019) Transcription Restart Establishes Chromatin Accessibility after DNA Replication. *Molecular cell*, **75**, 284-297.e286.
36. Zhu, C., Yu, M., Huang, H., Juric, I., Abnousi, A., Hu, R., Lucero, J., Behrens, M.M., Hu, M. and Ren, B. (2019) An ultra high-throughput method for single-cell joint analysis of open chromatin and transcriptome. *Nature structural & molecular biology*.
37. Kaya-Okur, H.S., Wu, S.J., Codomo, C.A., Pledger, E.S., Bryson, T.D., Henikoff, J.G. and Ahmad, K. (2019) CUT&Tag for efficient epigenomic profiling of small samples and single cells. *Elife*, **10**, 1930.
38. Carter, B., Ku, W.L., Kang, J.Y., Hu, G., Perrie, J., Tang, Q. and Zhao, K. (2019) Mapping histone modifications in low cell number and single cells using antibody-guided chromatin tagmentation (ACT-seq). *Nature communications*, **10**.
39. Wang, Q., Xiong, H., Ai, S., Yu, X., Liu, Y., Zhang, J. and He, A. (2019) CoBATCH for High-Throughput Single-Cell Epigenomic Profiling. *Molecular cell*, **76**, 206-216 e207.
40. Ai, S., Xiong, H., Li, C.C., Luo, Y., Shi, Q., Liu, Y., Yu, X., Li, C. and He, A. (2019) Profiling chromatin states using single-cell itChIP-seq. *Nature cell biology*, **21**, 1164-1172.
41. Liu, L., Liu, C., Quintero, A., Wu, L., Yuan, Y., Wang, M., Cheng, M., Leng, L., Xu, L., Dong, G. *et al.* (2019) Deconvolution of single-cell multi-omics layers reveals regulatory heterogeneity. *Nature communications*, **10**, 470.
42. Spektor, R., Tippens, N.D., Mimoso, C.A. and Soloway, P.D. (2019) methyl-ATAC-seq measures DNA methylation at accessible chromatin. *Genome research*, **29**, 969-977.
43. Di, L., Fu, Y., Sun, Y., Li, J., Liu, L., Yao, J., Wang, G., Wu, Y., Lao, K., Lee, R.W. *et al.* (2020) RNA sequencing by direct tagmentation of RNA/DNA hybrids. *Proceedings of the National Academy of Sciences of the United States of America*, **117**, 2886-2893.
44. Lu, B., Dong, L., Yi, D., Zhang, M., Zhu, C., Li, X. and Yi, C. (2020) Transposase-assisted tagmentation of RNA/DNA hybrid duplexes. *Elife*, **9**.
45. Xing, Q.R., Farran, C.A.E., Zeng, Y.Y., Yi, Y., Warrier, T., Gautam, P., Collins, J.J., Xu, J., Droge, P., Koh, C.G. *et al.* (2020) Parallel bimodal single-cell sequencing of transcriptome and chromatin accessibility. *Genome research*, **30**, 1027-1039.
46. Lareau, C.A., Ludwig, L.S., Muus, C., Gohil, S.H., Zhao, T., Chiang, Z., Pelka, K., Verboon, J.M., Luo, W., Christian, E. *et al.* (2020) Massively parallel single-cell mitochondrial DNA genotyping and chromatin profiling. *Nature biotechnology*.
47. Barnett, K.R., Decato, B.E., Scott, T.J., Hansen, T.J., Chen, B., Attalla, J., Smith, A.D. and Hodges, E. (2020) ATAC-Me Captures Prolonged DNA Methylation of Dynamic Chromatin Accessibility Loci during Cell Fate Transitions. *Molecular cell*, **77**, 1350-1364.e1356.
48. Zhang, C., Xu, Z., Yang, S., Sun, G., Jia, L., Zheng, Z., Gu, Q., Tao, W., Cheng, T., Li, C. *et al.* (2020) tagHi-C Reveals 3D Chromatin Architecture Dynamics during Mouse Hematopoiesis. *Cell reports*, **32**.
49. Ma, S., Zhang, B., LaFave, L.M., Earl, A.S., Chiang, Z., Hu, Y., Ding, J., Brack, A., Kartha, V.K., Tay, T. *et al.* (2020) Chromatin Potential Identified by Shared Single-Cell Profiling of RNA and Chromatin. *Cell*.
50. Liu, B., Xu, Q., Wang, Q., Feng, S., Lai, F., Wang, P., Zheng, F., Xiang, Y., Wu, J., Nie, J. *et al.* (2020) The landscape of RNA Pol II binding reveals a stepwise transition during ZGA. *Nature*.
51. Henikoff, S., Henikoff, J.G., Kaya-Okur, H.S. and Ahmad, K. (2020) Efficient chromatin accessibility mapping in situ by nucleosome-tethered tagmentation. *Elife*, **9**.
52. Payne, A.C., Chiang, Z.D., Reginato, P.L., Mangiameli, S.M., Murray, E.M., Yao, C.C., Markoulaki, S., Earl, A.S., Labade, A.S., Jaenisch, R. *et al.* (2020) In situ genome sequencing resolves DNA sequence and structure in intact biological samples. *Science*.
53. Xing, D., Tan, L., Chang, C.H., Li, H. and Xie, X.S. (2021) Accurate SNV detection in single cells by transposon-based whole-genome amplification of complementary strands. *Proceedings of the National Academy of Sciences of the United States of America*, **118**.

54. Lee, J.D., Paulo, J.A., Posey, R.R., Mugoni, V., Kong, N.R., Cheloni, G., Lee, Y.-R., Slack, F.J., Tenen, D.G., Clohessy, J.G. *et al.* (2021) Dual DNA and protein tagging of open chromatin unveils dynamics of epigenomic landscapes in leukemia. *Nature methods*.
55. Zhu, C., Zhang, Y., Li, Y.E., Lucero, J., Behrens, M.M. and Ren, B. (2021) Joint profiling of histone modifications and transcriptome in single cells from mouse brain. *Nature methods*.
56. Wang, Y., Lu, T., Sun, G., Zheng, Y., Yang, S., Zhang, H., Hao, S., Liu, Y., Ma, S., Zhang, H. *et al.* (2019) Targeting of apoptosis gene loci by reprogramming factors leads to selective eradication of leukemia cells. *Nature communications*, **10**, 5594.
57. Gray, L.T., Yao, Z.Z., Nguyen, N.T., Kim, T.K., Zeng, H.K. and Tasic, B. (2017) Layer-specific chromatin accessibility landscapes reveal regulatory networks in adult mouse visual cortex. *Elife*, **6**.
58. Pastor, W.A., Stroud, H., Nee, K., Liu, W., Pezic, D., Manakov, S., Lee, S.A., Moissiard, G., Zamudio, N., Bourc'his, D. *et al.* (2014) MORC1 represses transposable elements in the mouse male germline. *Nature communications*, **5**, 5795.
59. Senft, A.D., Costello, I., King, H.W., Mould, A.W., Bikoff, E.K. and Robertson, E.J. (2018) Combinatorial Smad2/3 Activities Downstream of Nodal Signaling Maintain Embryonic/Extra-Embryonic Cell Identities during Lineage Priming. *Cell reports*, **24**, 1977-1985 e1977.
60. Castro, J.P., Yancoskie, M.N., Marchini, M., Belohlavy, S., Hiramatsu, L., Kucka, M., Beluch, W.H., Naumann, R., Skuplik, I., Cobb, J. *et al.* (2019) An integrative genomic analysis of the Longshanks selection experiment for longer limbs in mice. *Elife*, **8**.
61. Onimaru, K., Marcon, L., Musy, M., Tanaka, M. and Sharpe, J. (2016) The fin-to-limb transition as the re-organization of a Turing pattern. *Nature communications*, **7**, 11582.
62. Snyder, M.P., Gingeras, T.R., Moore, J.E., Weng, Z., Gerstein, M.B., Ren, B., Hardison, R.C., Stamatoyannopoulos, J.A., Graveley, B.R., Feingold, E.A. *et al.* (2020) Perspectives on ENCODE. *Nature*, **583**, 693-698.
63. Bonkhofer, F., Rispoli, R., Pinheiro, P., Krecsmarik, M., Schneider-Swales, J., Tsang, I.H.C., de Bruijn, M., Monteiro, R., Peterkin, T. and Patient, R. (2019) Blood stem cell-forming haemogenic endothelium in zebrafish derives from arterial endothelium. *Nature communications*, **10**, 3577.
64. Bozek, M., Cortini, R., Storti, A.E., Unnerstall, U., Gaul, U. and Gompel, N. (2019) ATAC-seq reveals regional differences in enhancer accessibility during the establishment of spatial coordinates in the Drosophila blastoderm. *Genome research*, **29**, 771-783.
65. Daugherty, A.C., Yeo, R.W., Buenrostro, J.D., Greenleaf, W.J., Kundaje, A. and Brunet, A. (2017) Chromatin accessibility dynamics reveal novel functional enhancers in C. elegans. *Genome research*, **27**, 2096-2107.
66. Toenhake, C.G., Fraschka, S.A., Vijayabaskar, M.S., Westhead, D.R., van Heeringen, S.J. and Bartfai, R. (2018) Chromatin Accessibility-Based Characterization of the Gene Regulatory Network Underlying Plasmodium falciparum Blood-Stage Development. *Cell host & microbe*, **23**, 557-569 e559.
67. Ruiz, J.L., Tena, J.J., Bancells, C., Cortes, A., Gomez-Skarmeta, J.L. and Gomez-Diaz, E. (2018) Characterization of the accessible genome in the human malaria parasite Plasmodium falciparum. *Nucleic acids research*, **46**, 9414-9431.
68. Lu, Z., Marand, A.P., Ricci, W.A., Ethridge, C.L., Zhang, X. and Schmitz, R.J. (2019) The prevalence, evolution and chromatin signatures of plant regulatory elements. *Nat Plants*, **5**, 1250-1259.
69. Maher, K.A., Bajic, M., Kajala, K., Reynoso, M., Pauluzzi, G., West, D.A., Zumstein, K., Woodhouse, M., Bubb, K., Dorrity, M.W. *et al.* (2018) Profiling of Accessible Chromatin Regions across Multiple Plant Species and Cell Types Reveals Common Gene Regulatory Principles and New Control Modules. *The Plant cell*, **30**, 15-36.
70. Lu, Z., Hofmeister, B.T., Vollmers, C., DuBois, R.M. and Schmitz, R.J. (2017) Combining ATAC-seq with nuclei sorting for discovery of cis-regulatory regions in plant genomes. *Nucleic acids research*, **45**, e41.
